# Aromatic Higher Alcohols in Wine: Implication on Aroma and Palate Attributes during Chardonnay Aging

**DOI:** 10.3390/molecules26164979

**Published:** 2021-08-17

**Authors:** Antonio G. Cordente, Damian Espinase Nandorfy, Mark Solomon, Alex Schulkin, Radka Kolouchova, Ian Leigh Francis, Simon A. Schmidt

**Affiliations:** The Australian Wine Research Institute, P.O. Box 197, Glen Osmond, SA 5064, Australia; damian.espinasenandorfy@awri.com.au (D.E.N.); mark.solomon@awri.com.au (M.S.); alex.schulkin@awri.com.au (A.S.); radka.kolouchova@awri.com.au (R.K.); Leigh.francis@awri.com.au (I.L.F.); simon.schmidt@awri.com.au (S.A.S.)

**Keywords:** amino acid, yeast, wine, sulfur, aroma, aging, QDA

## Abstract

The higher alcohols 2-phenylethanol, tryptophol, and tyrosol are a group of yeast-derived compounds that have been shown to affect the aroma and flavour of fermented beverages. Five variants of the industrial wine strain AWRI796, previously isolated due to their elevated production of the ‘rose-like aroma’ compound 2-phenylethanol, were characterised during pilot-scale fermentation of a Chardonnay juice. We show that these variants not only increase the concentration of 2-phenylethanol but also modulate the formation of the higher alcohols tryptophol, tyrosol, and methionol, as well as other volatile sulfur compounds derived from methionine, highlighting the connections between yeast nitrogen and sulfur metabolism during fermentation. We also investigate the development of these compounds during wine storage, focusing on the sulfonation of tryptophol. Finally, the sensory properties of wines produced using these strains were quantified at two time points, unravelling differences produced by biologically modulating higher alcohols and the dynamic changes in wine flavour over aging.

## 1. Introduction

*Saccharomyces cerevisiae* performs a wide range of industrial fermentations that functionally depend upon its ability to convert sugars to ethanol and carbon dioxide efficiently. While performing this primary function, *S. cerevisiae* also produces a range of secondary metabolites, such as esters, volatile fatty acids, higher alcohols, and volatile sulfur compounds (VSCs), which contribute substantially to the flavour and aroma of wine [1], beer [2], and sake [3]. Of these fermentation compounds, higher alcohols and esters are the most abundant groups [1].

Higher alcohols, also known as fusel alcohols, are compounds with more than two carbon atoms. These alcohols are derived from yeast amino acid metabolism via the Ehrlich pathway [4]. Amino acids assimilated by the Ehrlich pathway include the aliphatic or branched-chain (leucine, valine and isoleucine) and aromatic (phenylalanine, tyrosine and tryptophan) amino acids, as well as the sulfur-containing amino acid methionine. The Ehrlich pathway consists of three steps: the initial transamination of the amino acid to the corresponding α-keto acid analogue, decarboxylation to an aldehyde, and reduction to the corresponding higher alcohol by an alcohol dehydrogenase [4]. While the metabolism of aliphatic and aromatic amino acids via the Ehrlich pathway has been extensively studied in yeast, little is known about the branch of the pathway involved in methionine catabolism. However, some similarities exist between the catabolism of methionine and that of the aromatic amino acids. The aminotransferases Aro8p and Aro9p, which catalyse the first step of the Ehrlich pathway for aromatic amino acids, also play an essential role in methionine transamination [5,6]. Similarly, the broad-substrate phenylpyruvate decarboxylase Aro10p is also involved in the second step of the Ehrlich pathway for methionine [7,8].

Except for 2-phenylethanol (2-PE), which is derived from phenylalanine and associated with a ‘rose-like’ odour quality [9], the individual contribution of higher alcohols to wine aroma is not considered to be pleasant, particularly at higher concentrations [10]. For example, the higher alcohols derived from branched-chain amino acids (2-methylpropanol, 3-methylbutanol and 2-methylbutanol) are associated with ‘solvent’ and ‘fusel’ aroma descriptors, while 3-methylthio-1-propanol (or methionol), derived from methionine, imparts a ‘boiled or cooked potato’ aroma in wine [10]. Aside from these negative associations, higher alcohols may also contribute to wines’ overall ‘vinous’ aroma as part of the ‘aroma buffer’ [11]. Of the many higher alcohols, tyrosol (TyrOH) and tryptophol (TOL) (from tyrosine and tryptophan, respectively) have not been associated with any aroma descriptors. However, there is growing evidence that they influence in-mouth sensory properties, especially the taste of some fermented beverages, as both compounds have been associated with bitterness in wine, sake, and beer [12,13,14]. Recently, it has been reported that TOL can react with sulfur dioxide (SO_2_), which is widely used in winemaking as an additive due to its antimicrobial and antioxidant effects, to yield the tryptophol-2-sulfonate (TOL-SO_3_H) adduct [15]. This reaction is favoured by the presence of small amounts of oxygen in wine [15], and the equilibrium towards the formation of TOL-SO_3_H from TOL seems to be increased by bottle aging, particularly for white wines [16]. Although the effect of TOL-SO_3_H on the taste and mouthfeel properties of fermented beverages remains unclear, it has recently been linked with bitterness [17].

Higher alcohols are substrates for acetate ester production, a reaction catalysed by yeast alcohol acetyltransferases. Many acetate esters are associated with ’fruity’ and ‘floral’ aromas in wine [18]. For example, both 2-PE and 3-methylbutanol (‘solvent’) can be esterified and converted into 2-phenylethyl acetate (2-PEA) (‘rose’, ‘fruity’, ‘honey’) and 3-methylbutyl acetate (‘banana’), respectively. Acetate esters are important contributors to the aroma of young wines, as their concentration tends to decrease post-fermentation with wine storage due to non-enzymatic, acid-catalysed reactions in the wine matrix [19,20].

Due to the ability of 2-PE and 2-PEA to impart floral notes, these compounds present an opportunity to shape wine style. The ability to influence the production of 2-PE and 2-PEA in white wines would be beneficial because, although naturally occurring, they are usually present at relatively low concentrations and are unlikely to impart a definitive character [1,21]. The concentration of amino acids in grape must is a crucial factor influencing the production of higher alcohols by yeast: increased concentration of a specific precursor amino acid will usually increase the concentration of the corresponding higher alcohol [22]. Therefore, one widely used strategy to increase the formation of 2-PE is to select yeast strains that overproduce the precursor phenylalanine. Obtaining phenylalanine-overproducing yeast can be achieved by selecting strains resistant to toxic fluorinated analogues of phenylalanine. Such a strategy has been successfully used to generate industrial *S. cerevisiae* strains that improve the organoleptic properties of sake, wine, and bread [23,24,25].

Conversely, and with the exception of a small group of polyfunctional thiols associated with ‘fruity’ and ‘tropical’ characters [26], most VSCs generated by yeast are considered to be off-flavours in wine, particularly the ‘rotten-egg’-imparting compound hydrogen sulfide (H_2_S). Similarly, a range of compounds derived from methionine by either enzymatic or non-enzymatic reactions are also associated with negative attributes in wine, such as methanethiol (MeSH) (‘sewage and rubber’ aromas), methional (‘cooked potato’ aroma) and methyl thioacetate (MeSAc) (‘sulfurous and cheesy’ aromas) [6,8,27]. While the formation of H_2_S by yeast has been extensively studied, and commercial low H_2_S producing strains have been generated [28], little is known about the formation of odoriferous VSCs derived from methionine, and few strategies exist to reduce their formation during fermentation.

This paper explores the influence of compounds derived from the metabolism of both aromatic and sulfur-containing amino acids in the sensory profile of Chardonnay wine over time. For this purpose, we characterised five variants of a commercially available wine yeast strain (AWRI796), which were previously isolated because of their high 2-PE production phenotype. Here we show that these variants influence not only the production of 2-PE and 2-PEA but also the concentration of the higher alcohols TOL, TyrOH, and methionol, as well as other odoriferous VSCs derived from the amino acid methionine. The progression of these compounds and the equilibrium shift between TOL-SO_3_H and TOL during wine ageing are also reported. Formal sensory analysis was conducted on the Chardonnay wines produced by each strain at two time points, and the links between the resulting changes to chemical composition and sensory properties are presented and explored.

## 2. Results

### 2.1. Pilot-Scale White Winemaking of Five 2-Phenylethanol Overproducing Strains

Previously, we isolated a range of variants from the commercial wine yeast AWRI796 that were resistant to toxic fluorinated analogues of phenylalanine. These variants were shown to overproduce 2-PE and 2-PEA to different extents in laboratory-scale fermentations [23]. A single variant, AWRI2940, was further characterised in a Chardonnay pilot-scale winemaking study, where its high 2-PE production phenotype was validated. Sensory evaluation of the wines showed that while an increased ‘floral’ aroma was the attribute most affected by AWRI2940, this variant also produced wines that were noted as having a more ‘bitter’ taste and ‘astringent’ mouthfeel than the parent strain [23]. Compositional analysis showed that AWRI2940 produced a higher concentration of TOL and a lower concentration of TyrOH than the parent strain, compounds that have been associated with bitterness in different alcoholic beverages [12,13,29,30].

**Figure 1:**
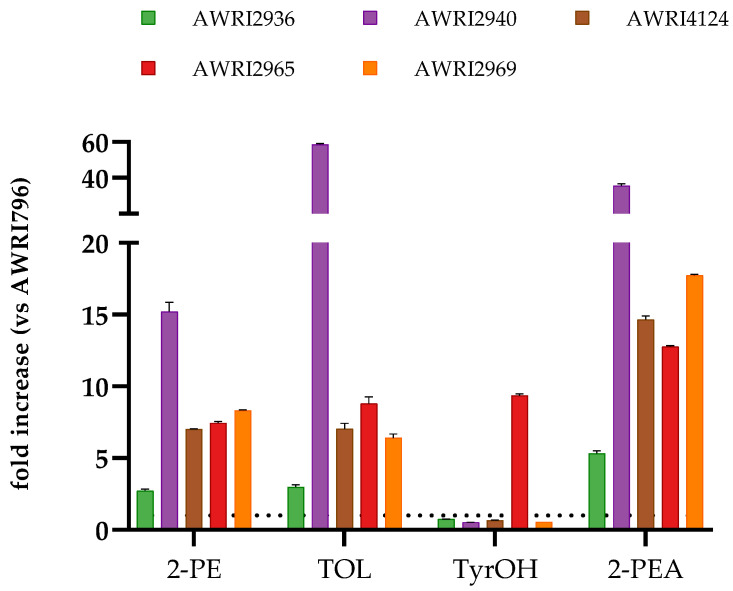
Production of the higher alcohols 2-PE, TOL and TyrOH, and 2-PEA, in Chardonnay wines by five variants of the wine strain AWRI796 carrying mutations in Aro4p (AWRI2965) or Tyr1p (AWRI2936, 2940, 4124, and 2969). Results are expressed as the average fold change in the concentration of these metabolites relative to the control strain AWRI796 (indicated with a dashed line) after alcoholic fermentation. Error bars show the standard deviation of three independent fermentations.

The effects of variations in 2-PE, TyrOH and TOL concentration on wine sensory properties were determined using five mutants of AWRI796 together with parent strain (control) to produce Chardonnay wine. The variant strains harbour distinct mutations in two of the enzymes involved in aromatic amino acid metabolism: Tyr1p and Aro4p (summarised in Table S1). The 19 L Chardonnay ferments were conducted in triplicate and were complete after 31 days. Post-fermentation analysis of the volatiles confirmed the 2-PE overproduction phenotype for all five variants (Figure 1 and Table S2). Fermentation with strain AWRI2940 resulted in a 15-fold increase in the concentration of 2-PE relative to the parent AWRI796. The strains AWRI2965, AWRI2969 and AWRI4124 showed a more moderate 2-PE overproduction phenotype (between 7- and 8-fold increase). AWRI2936 was the lowest 2-PE producer of all the variants (3-fold increase). In all five mutants of AWRI796, the relative increases of 2-PEA were even higher than those observed for 2-PE, ranging from 6- to nearly 40-fold (Figure 1). As expected, the concentration of 2-PE and 2-PEA was highly correlated (r = 0.983, *p* < 0.0001, Figure S1).

**Figure 2:**
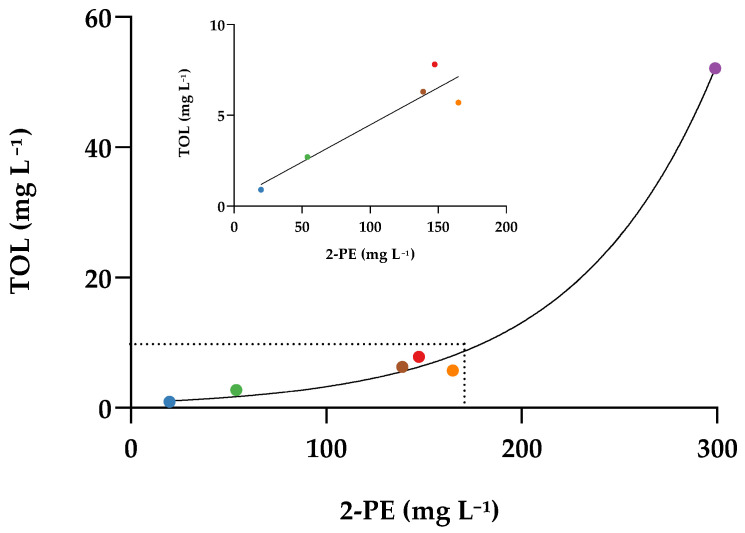
Relationship between 2-PE and TOL concentrations (mg L^−1^) at the end of alcoholic fermentation in Chardonnay wines. The wines were fermented by the parent strain AWRI796 (●), and five variants carrying mutations in Aro4p (AWRI2965 (●)) or Tyr1p (AWRI2936 (●), AWRI2940 (●), AWRI4124 (●) and AWRI2969 (●)). The area under the dotted lines (inset) highlights the linear relationship between these two higher alcohols at lower concentrations.

All five yeast variants, particularly AWRI2940, produced more TOL than AWRI796 (Figure 1). TOL was also positively correlated with the 2-PE concentration. The relationship was linear for the parent and the four variants that produced low to moderate concentrations of TOL (R^2^ = 0.87, *p* < 0.0001), while an exponential model was a better fit when all strains were included (R^2^ = 0.97) (Figure 2). In contrast to TOL production, the four Tyr1p variants produced lower concentrations of TyrOH than the parent AWRI796, while the Aro4p variant (AWRI2965) produced substantially more (9-fold increase) (Figure 1). While there was no evidence for strain-based differences in the concentration of branched-chain amino acid derived higher alcohols, there was evidence for strain-dependent differences in the concentration of the respective acetate esters (Table S2). Notably, the concentration of ethyl acetate, associated with a ‘nail polish remover’ aroma, was not affected by any of the 2-PE overproducing variants.

### 2.2. Effect of Wine Aging on TOL/TOL-SO_3_H Equilibrium

**Figure 3:**
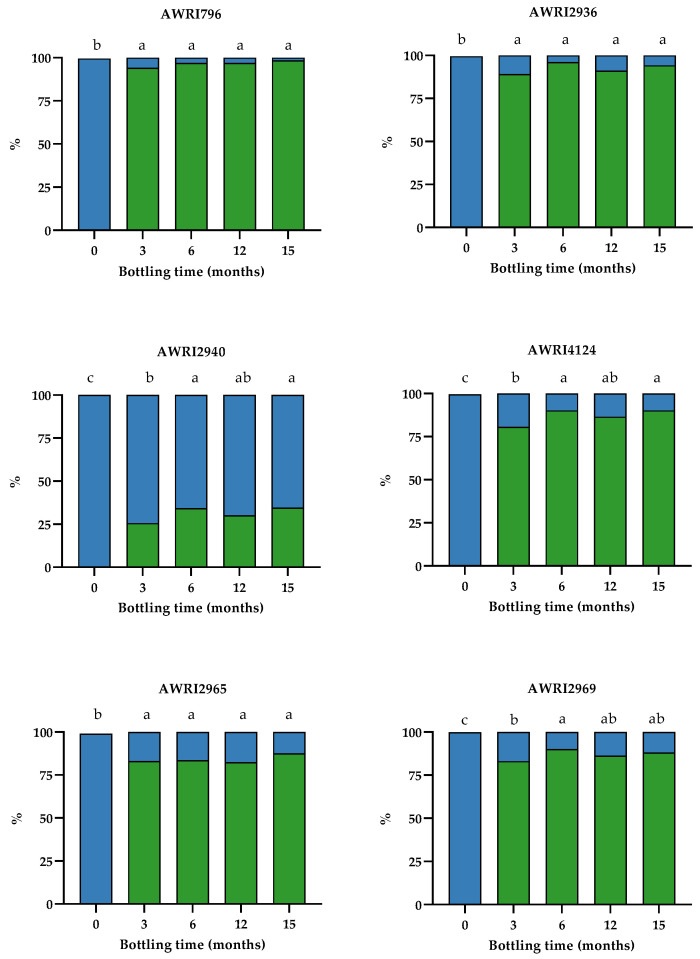
Relative molar percentages of TOL (blue) and TOL-SO_3_H (green) in relation to bottling age in Chardonnay wines made with the parent AWRI796 and five variants carrying mutations in Aro4p (AWRI2965) or Tyr1p (AWRI2936, 2940, 4124, and 2969). Means with the same letters are not significantly different from each other (Tukey’s test, alpha = 0.05).

We assessed the effect of bottle storage duration on the concentration of aromatic higher alcohols, focusing on the sulfonation of TOL to yield the TOL-SO_3_H adduct. At the end of alcoholic fermentation, only wines made with the highest TOL producer (AWRI2940) contained TOL-SO_3_H (0.26 mg L^−1^), representing a 0.3% molar conversion of TOL into TOL-SO_3_H. The post-fermentation concentration of total SO_2_ in the wines averaged 21 mg L^−1^, with no detectable free SO_2_ (Table S2). On completion of alcoholic fermentation, 80 mg L^−1^ of SO_2_ was added to the wines, followed by a lengthy period of cold-stabilisation (two months). Before bottling, free SO_2_ concentration was adjusted to between 35 and 40 mg L^−1^ (Table S3). Conversion of a substantial percentage of TOL into its sulfonated adduct was evident after three months in-bottle: for the low and moderate TOL producers (<10 mg L^−1^ TOL) the yield of TOL-SO_3_H was at least 80%, while a 26% yield was observed in wines made using the high TOL producer (AWRI2940) (Figure 3).

After six months in-bottle, an equilibrium between TOL and TOL-SO_3_H species was reached, with longer storage times (12 and 15 months) having little or no effect on the conversion of the higher alcohol into the sulfonated adduct (Figure 3). The maximum molar % yield of TOL-SO_3_H inversely correlated with the concentration of TOL at the end of alcoholic fermentation (r = −0.998, *p* < 0.0001) (Figure S2). For the control strain (AWRI796 strain), which only produced 0.89 mg L^−1^ of TOL, almost 100% of the available TOL had been converted into TOL-SO_3_H. For the four moderate TOL producers, the % yield of TOL-SO_3_H ranged from 88 to 94%, while for the high TOL producer (AWRI2940) the yield was only 36% (Figure 3).

**Figure 4:**
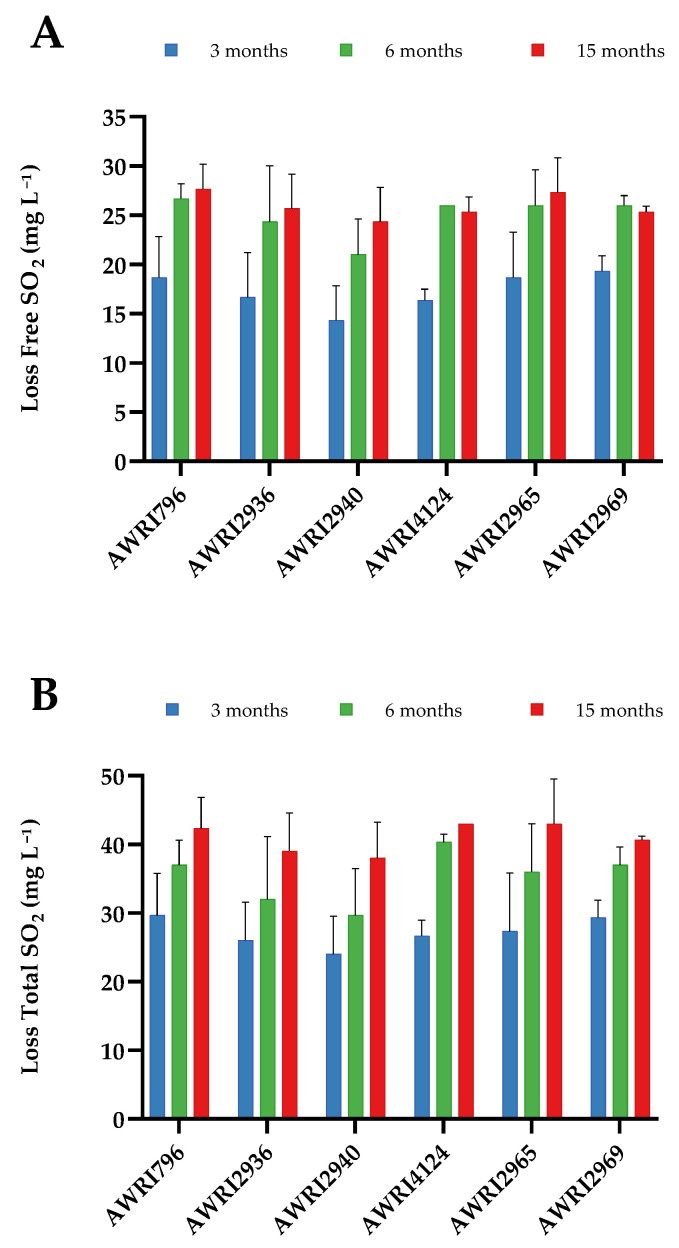
Loss in free (**A**) and total (**B**) SO_2_ concentrations (mg L^−1^) during storage in Chardonnay wines made with the parent AWRI796 and five variants carrying mutations in Aro4p (AWRI2965) or Tyr1p (AWRI2936, 2940, 4124, and 2969). Wines analysed after 3 (blue), 6 (green), and 15 (red) months of storage at 15 °C were compared to the respective SO_2_ concentrations on the day of bottling (which averaged 39 and 110 mg L^−1^ of free and total SO_2_, respectively). Results are expressed as the mean and standard deviation of three independent fermentations. No differences between strains were seen at any of the three storage times assessed (Tukey’s test, alpha = 0.05).

We sought to determine whether TOL-SO_3_H formation was a significant contributor to SO_2_ loss during bottle storage. On the day of bottling, the concentration of free SO_2_ averaged 39 mg L^−1^ across all wines (Table S3). In the case of AWRI2940, we would expect a decrease in SO_2_ concentration of 7.6 mg L^−1^ related to adduct formation assuming an equimolar reaction between SO_2_ and TOL. For the low and moderate producers, we would expect less than 3 mg L^−1^ of SO_2_ to be consumed. Measured decreases in SO_2_ concentration were similar across all the wines after three months in-bottle: decreases of between 14 and 19 mg L^−1^ in free SO_2_ concentration and between 24 and 30 mg L^−1^ in total SO_2_ concentration were observed. Longer storage times resulted in further losses of SO_2_ with respect to pre-bottling concentrations, with the data offering no evidence for an effect of strain on the loss of free or total SO_2_ during wine aging (Figure 4). No significant correlation between loss of SO_2_ and TOL-SO_3_H concentration was observed at any time point assessed (Figure S3).

### 2.3. Effect of Wine Ageing on Volatile Sulfur Compounds

The effects of alterations to aromatic amino acid metabolism in yeast on the formation of several VSCs derived from methionine were also investigated in the Chardonnay wines after 3 and 15 months in-bottle. In yeast, two competing pathways are involved in methionine degradation: the Ehrlich and demethiolation pathways [31]. In the Ehrlich pathway, methionine is transaminated and decarboxylated to methional, and subsequently reduced to the higher alcohol methionol [4,6]. Methionine is also the precursor of MeSH, the production of which can occur enzymatically via demethiolation [8], or non-enzymatically [32]. Therefore, the question arises: how do alterations to aromatic amino acid metabolism change the contribution of these two methionine degradation pathways to wine composition?

**Figure 5:**
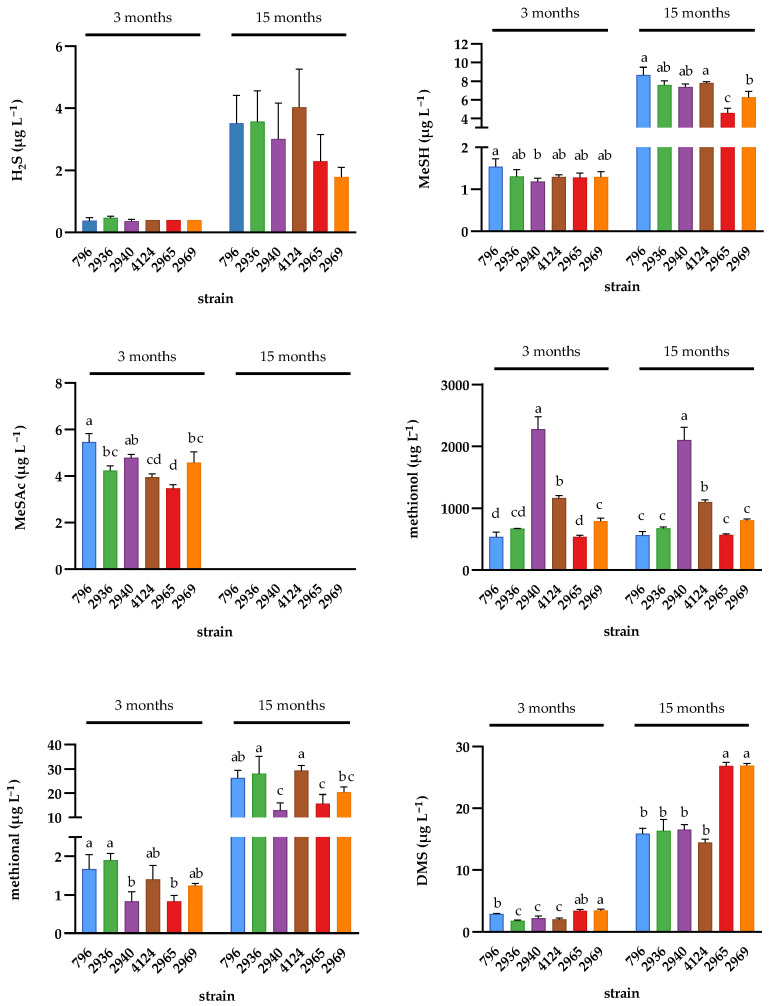
Concentrations (in µg L^−1^) of VSCs in Chardonnay wines made with the parent AWRI796 and five variants carrying mutations in Aro4p (AWRI2965) or Tyr1p (AWRI2936, 2940, 4124, and 2969) at two different time points during wine storage. The results are expressed as the mean and standard deviation of three independent replicates. ANOVAs were conducted separately for each time point. Means with the same letters are not significantly different from each other (Tukey’s test, alpha = 0.05). Concentrations of MeSAc at the 15 month time point were below the limit of quantification of the technique (<5 µg L^−1^).

After three months, the concentration of methionol was greater in wines made with the high 2-PE producing strains (AWRI4124, AWRI2969 and AWRI2940) than those made with the parent strain (AWRI796), whereas the low 2-PE producer and the high TyrOH-producing strains (AWRI2936 and AWRI2965, respectively) accumulated similar concentrations of methionol to the parent (Figure 5). The concentrations of methional were lowest in wines produced by strains AWRI2940 and AWRI2965. Similar trends were observed in the concentration of compounds derived from the demethiolation pathway; the concentrations of MeSH and its thioacetate, MeSAc, were both lower in wines made with AWRI2940 and AWRI2965.

After 15 months in-bottle, the concentration of most VSCs had increased in all wines relative to their concentration at three months. Nevertheless, a similar strain profile was evident in the VSC concentrations of wines sampled at both time points. Wines made with strain AWRI2965 still had a significantly lower concentration of MeSH and methional than the parent strain (AWRI796) while showing a slightly elevated concentration of dimethylsulfide (DMS). No statistical evidence supporting differences between the strains in the concentration of H_2_S was observed at any time point (Figure 5).

**Figure 6:**
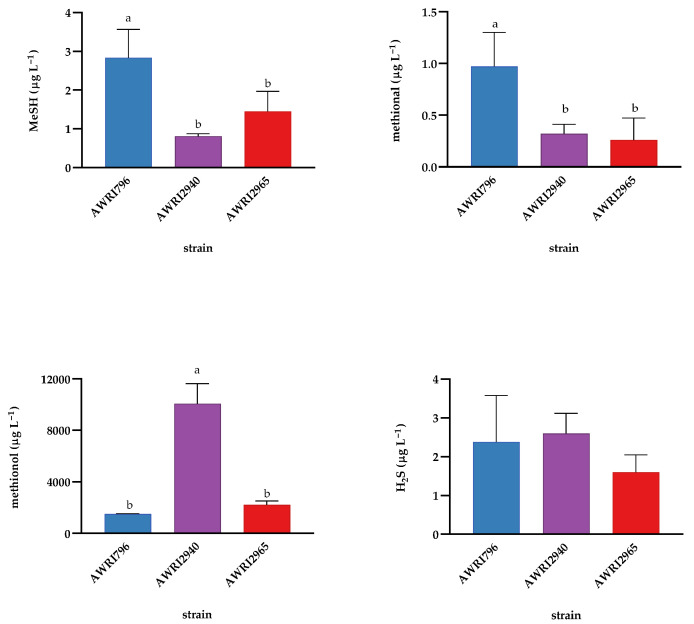
Concentrations (in µg L^−1^) of VSCs after fermentation of a synthetic grape medium (SGM). Fermentations were carried out with the parent AWRI796, and two variants carrying mutations in either Aro4p (AWRI2965) or Tyr1p (AWRI2940). The results are expressed as the mean and standard deviation of three independent replicates. Means with the same letters are not significantly different from each other (Tukey’s test, alpha = 0.05). No DMS and MeSAc were detected.

To further confirm the effects that mutations in either Tyr1p or Aro4p might have on methionine catabolism, two of the variants were used to ferment a synthetic grape medium (SGM) with grape-like concentrations of methionine [33]. The two strains used were characterised by moderate (AWRI2965:Aro4) and high (AWRI2940:Tyr1) 2-PE production, thus reflecting different levels of activation of the Ehrlich pathway (Figure S4). Results in SGM reflected those found in the Chardonnay wines (Figure 6). Fermentation with both variants resulted in a lower concentration of MeSH and methional relative to the parent AWRI796. Conversely, AWRI2940 produced substantially more of the Ehrlich pathway end product methionol than the other strains.

### 2.4. Quantitative Descriptive Sensory Analysis

Sensory descriptive analysis of the wines was performed twice, after 3 and 15 months in-bottle. The analyses were compared to assess the effect of bottle storage on wine sensory attributes. The mean scores for a subset of sensory attributes are summarised in Table 1 and Table 2. The sensory differences between the samples were relatively subtle after three months, indicated by the small ANOVA F-ratios (Table S4). Statistical evidence (*p* < 0.05) supports differences between strains in three attributes rated by the panel: ‘yellow colour intensity’, ‘floral aroma’ and ‘grassy flavour’ (Table 1 and Table S5). Wines from strain AWRI2940 (the highest 2-PE producer) were rated highest in both ‘floral aroma’ and ‘yellow colour’, but none of the variants were rated higher than the parent (AWRI796) in ‘floral aroma’. Weak evidence (*p* < 0.15) alluded to possible trends among the strains for the attributes ‘grassy aroma’, ‘cooked vegetable/potato aroma’, ‘sweetness’, ‘bitterness’, ‘stone fruit flavour’ and ‘flint flavour’. In particular, wines made with AWRI796 and AWRI4124 were rated highly in the attributes ‘cooked vegetable/potato aroma’ and ‘sweetness’, while there was a trend for ‘bitterness’ to be rated lowest for the low TOL-/TOL-SO_3_H-producing strains AWRI2936 and AWRI796 compared to the other strains.

After 15 months in-bottle, differences in wine sensory profiles were more apparent, highlighted by more robust statistical evidence and larger F-ratios for more attributes in the ANOVA (Table S6). Seven attributes were influenced by the strains (Table 2 and Table S7). There was very strong evidence (*p* < 0.001) that strains produced wines with different intensities of ‘yellow colour’, ‘cooked vegetable/potato aroma’, ‘sourness’ and ‘sweetness’. There was evidence (*p* < 0.05) for differences in ‘pungency’, ‘rose aroma’ and ‘stone fruit flavour’. Notably, at this time point, judges chose to rate a specific ‘rose aroma’ quality rather than the more general ‘floral’ aroma attribute used to describe the wines at three months. Wine from strain AWRI2940 was notably higher in ‘rose aroma’, as well as in ‘yellow colour’ and ‘sourness’, while lower in ‘cooked vegetable/potato’ and ‘sweetness’. Strains AWRI2936, AWRI2965, and AWRI4124 were rated with intermediate values for ‘rose aroma’ (Table 2), compared to the lowest (AWRI796 parent) and highest (AWRI2940) strains. Wines made with yeast strains AWRI796 and AWRI4124 were rated highly in the ‘cooked vegetable/potato aroma’. 

### 2.5. Relationships between Chemical Composition and Sensory Data

**Figure 7:**
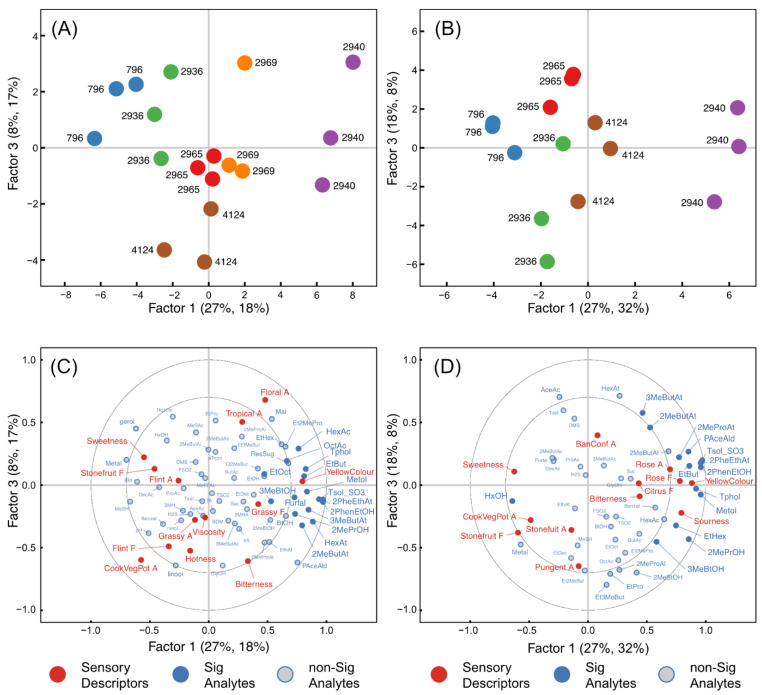
Factor 1 and 3 for the scores (**A**,**B**) and loadings (**C**,**D**) plots from PLS regression models for Chardonnay wines after 3 months (**A**,**C**) and 15 months (**B**,**D**) of aging generated using important sensory attributes (Y variables, red) and com-positional compounds (X variables, blue) for each fermentation replicate of the winemaking treatments. Compounds significant to each PLS model are indicated by filled blue circles and compounds with a lesser contribution to the model are shown as open circles. The proportion of the X-variance explained by the two factors is denoted by the first value in parentheses, the proportion of Y-variance by the second value. Chemical composition and labels are summarised in Tables S10 and S11.

Partial least squares regression (PLS-R) was used to investigate the relationships between wine composition and sensory attributes for both time points at which the wines were evaluated (Figure 7). Chemical compounds and sensory attributes situated together in Figure 7C,D are covariant, and attributes toward the outside of the plots were well modelled. Compounds that were significant contributors to the overall model are indicated (sig analytes), while the magnitude of their regression coefficient can identify compounds most implicated in a specific attribute.

Both the 3-month and 15-month PLS-R models indicated three optimum factors, explaining 50% and 54% of the variance of the sensory data, respectively. Figure 7 shows the scores (A,B) and loadings (C,D) plots for Factors 1 and 3. In both models, higher alcohols (2-PE, TOL, TOL-SO_3_H, methionol), and the compounds 2-PEA, ethyl butanoate, 2- and 3-methylbutyl acetate, 2-methylpropanol, 3-methylbutanol were significant contributors to the sensory differences and were heavily loaded on Factor 1.

Of the sensory attributes at the 3-month time-point, ‘yellow colour’ (R^2^ calibration 0.69 and R^2^ validation 0.51), ‘floral aroma’ (R^2^ calibration 0.67 and R^2^ validation 0.20), ‘cooked vegetable/potato aroma’ (R^2^ calibration 0.75 and R^2^ validation 0.27), ‘sweetness’ (R^2^ calibration 0.56 and R^2^ validation 0.28), ‘viscosity’ (R^2^ calibration 0.40 and R^2^ validation 0.20), and ‘stone fruit flavour’ (R^2^ calibration 0.59 and R^2^ validation 0.30) were relatively well modelled but not so well predicted, indicated by the low R^2^ validation. TOL and TOL-SO_3_H were significantly associated with ‘yellow colour’. No compounds were significant for ‘floral aroma’ but two monoterpenes, *cis*-rose oxide and α-terpineol, and several esters had moderately sized positive regression coefficients (values > 0.04) for this attribute, while 2-PE and 2-PEA were only weakly positively associated. ‘Cooked vegetable/potato aroma’ was associated with several sulfur compounds, notably H_2_S and MeSH. ‘Bitterness’ was most strongly related to volatile acidity and 2-phenylacetaldehyde, with TyrOH and TOL-SO_3_H weakly associated. TOL-SO_3_H and volatile acidity, in addition to 2-PE and 2-PEA, were significantly negatively associated with ‘sweetness’.

In the 15-month PLS model, similar links between compounds and sensory attributes emerged, but this model was generally stronger and predicted most attributes well. From the three-factor model, ‘yellow colour’ (R^2^ calibration 0.83 and R^2^ validation 0.67), ‘stone fruit aroma’ (R^2^ calibration 0.55 and R^2^ validation 0.22), ‘rose aroma’ (R^2^ calibration 0.51 and R^2^ validation 0.07), ‘cooked vegetable/potato aroma’ (R^2^ calibration 0.76 and R^2^ validation 0.59), ‘sweetness’ (R^2^ 0.39 calibration and R^2^ validation 0.23), ‘stone fruit flavour’ (R^2^ calibration 0.60 and R^2^ validation 0.34), ‘rose flavour’ (R^2^ calibration 0.62 and R^2^ validation 0.21), and ‘sourness’ (R^2^ calibration 0.69 and R^2^ validation 0.48) were relatively well modelled, while ‘banana confection aroma’, ‘citrus flavour’, ‘pungent aroma’, and ‘bitterness’ were not modelled well. Similar to the model at three months, TOL and TOL-SO_3_H were again significantly associated with ‘yellow colour’, together with several other compounds. Of the many compounds associated with ‘rose aroma and flavour’, 2-PE and 2-PEA had the largest positive regression coefficients, while the volatiles 2-phenylacetaldehyde, methionol, hexyl acetate, 2- and 3-methylbutyl acetate, ethyl butanoate, 2-methylpropyl acetate, ethyl 2-methyl propanoate, H_2_S, and non-volatiles TOL and TOL-SO_3_H were also associated with these attributes. There was strong evidence for an association between ‘cooked vegetable/potato aroma’ and the compounds methional (regression coefficient 0.09) and MeSH (regression coefficient 0.07); although the association with H_2_S was weak, it had a relatively high regression coefficient of 0.04. There was strong evidence for a negative association between the compounds TOL and TOL-SO_3_H and ‘sweetness’, and a positive association with ‘sourness’. Although ‘bitterness’ was not well modelled, TOL and TOL-SO_3_H were positively associated with this attribute, with relatively high regression coefficients.

## 3. Discussion

In this study, we characterised the chemical and sensory profiles of a group of five variants derived from the commercial wine strain AWRI796 in a pilot-scale winemaking trial in Chardonnay. These five yeast strains’ aromatic higher alcohol profiles were previously related to specific mutations found in two aromatic amino acid biosynthesis pathway genes, *ARO4* and *TYR1* [22]. The product of *ARO4* catalyses the first step in aromatic amino acid biosynthesis. It was shown that the Aro4p^Q166R^ mutation was responsible for the overproduction of 2-PE by AWRI2965 during laboratory-scale fermentation and the intracellular accumulation of the aromatic amino acids tryptophan, phenylalanine, and tyrosine [23]. We confirmed that AWRI2965 accumulates 2-PE in addition to high quantities of TyrOH while also producing slightly elevated concentrations of TOL compared to the strain from which it was derived (AWRI796). Together, these results indicate that the overall effect of Aro4p^Q166R^ could be to redirect pathway flux towards the accumulation of aromatic amino acids, and their respective higher alcohols, through the Ehrlich pathway.

The other four strains characterised in this work harboured point mutations in *TYR1,* a gene encoding a prephenate dehydrogenase enzyme catalysing the penultimate step in tyrosine biosynthesis. The higher alcohol profile of the *TYR1* mutant strains differed relative to the profiles of both AWRI2965 and AWRI796. While the *TYR1* mutant strains overproduced both TOL and 2-PE, they also produced significantly lower concentrations of TyrOH than the parent. It has been shown that Tyr1p mutations result in reduced formation of tyrosine and overproduction of 2-PE [23,25]. These results are compatible with a decrease in prephenate dehydrogenase activity, limiting tyrosine production, and consequently TyrOH biosynthesis. The constraint on the tyrosine branch of the aromatic amino acid biosynthesis pathway results in metabolic overflow into the tryptophan and phenylalanine branches, causing the increased production of these amino acids and their respective higher alcohols [23,25].

We observed dynamic changes between TOL and its sulfonated adduct, TOL-SO_3_H, during wine storage, an event associated with wine aging and promoted by small amounts of oxygen [15,16]. Recently, small amounts of TOL-SO_3_H were detected during laboratory-scale fermentation of a Chardonnay must with a yeast strain that produced high concentrations of TOL and SO_2_, indicating that sulfonation of TOL can also occur in anaerobic conditions [17]. Similarly, in our Chardonnay study, trace amounts of TOL-SO_3_H were found just after alcoholic fermentation, but only in the wines made with the highest TOL producer, AWRI2940. At this stage, the concentration of total SO_2_ in the wines averaged 21 mg L^−1^, with no detectable free SO_2_. Subsequently, after three months of storage at 15 °C, during which the wines had been in contact with high concentrations of externally added free SO_2_, a substantial amount of the initial TOL produced by yeast was converted into its sulfonated adduct.

Our results demonstrate that the time required to reach equilibrium between TOL and its sulfonated adduct, typically between 3 and 6 months, is independent of the initial amount of TOL. Longer storage times have little or no effect on the yield of TOL-SO_3_H. In wines made with low and moderate TOL producers, the maximum observed conversion percentage (>85%) is consistent with that reported in a range of commercial white wines of different ages [16]. The molar ratio between free SO_2_ and TOL was between 13 and 132 in wines with low to moderate TOL concentrations favouring the formation of TOL-SO_3_H. The high conversion yields are therefore not unexpected. In the wines made with the highest TOL producer, AWRI2940, this molar ratio was as low as 1.8, explaining the lower conversion yield. The TOL concentration found in Chardonnay wines fermented with the low and moderate TOL-producing strains was in the range of those reported in the literature for commercial white wines, typically below 5 mg L^−1^ [16,34,35]. At the TOL concentration found in commercial white wines, and even considering a total conversion into its sulfonated adduct, the expected loss of SO_2_ due to this reaction would be less than 2 mg L^−1^. A survey of a large number of commercial bottled Australian white wines [36] found an average of 31 mg L^−1^ of free SO_2_ in the most recent vintages assessed, a concentration similar to that found in our Chardonnay wines at bottling (39 mg L^−1^). These data suggest that the conversion of TOL into TOL-SO_3_H makes only a limited contribution to the losses of SO_2_ observed during wine development, which are primarily driven by reaction with dissolved O_2_ in wines after bottling and by sulfonation of other species [16,36]. This conclusion is supported by the lack of a correlation between TOL-SO_3_H concentration and SO_2_ loss in wine observed here.

Wines made with AWRI2940 accumulated exceptionally high concentrations of both TOL and TOL-SO_3_H. This high accumulation allowed us to assess the possible effects of TOL and TOL-SO_3_H on white wine sensory properties. After 15 months in-bottle, wines made with AWRI2940 were rated higher in ‘sourness’ and lower in ‘sweetness’, and tended to be more bitter for some assessors even though these wines had slightly elevated fructose and glycerol concentrations (both sweet compounds individually) relative to the control. The PLS-R results suggest that both TOL and TOL-SO_3_H may impart a degree of ‘bitterness’ to these wines while decreasing ‘sweetness’ and increasing ‘sourness’ ratings, probably through well-established taste–taste interactions [37], especially for wines produced with AWRI2940.

To our knowledge, no sensory recognition or detection thresholds for TOL-SO_3_H in a wine matrix have been published, but Van Gemert [38] lists a wide range for the detection threshold of TOL in beer (between 10 and >414 mg/L). Less clear is the possible effect of TyrOH on the sensory properties of white wines. Despite the potential of this higher alcohol to induce a ‘bitter finish’ in alcoholic beverages such as sake and beer [12,14,30], concentrations of TyrOH between its detection and recognition thresholds have been suggested to have a positive ‘taste-sharpening’ effect in sake [14,39]. Consequently, some effort has been devoted to breeding sake strains that overproduce this higher alcohol [14,40]. Different sensory thresholds for TyrOH appear in the literature: while a high threshold value (346 mg L^−1^) eliciting bitter taste was reported in water [29], lower and different detection thresholds have been reported for beer (between 20 and 100 mg L^−1^) [12]. Even though typical concentrations of TyrOH (20–30 mg L^−1^) have been reported to impart bitterness in wine [13], to the best of our knowledge, these claims are not supported by documented research. Sapis and Ribereau-Gayon [34] added 50 mg L^−1^ of TyrOH to white wine and observed no influence on sensory properties, and only when unrealistically high concentrations were added (500 mg L^−1^) did TyrOH seem to depress the overall ‘flavour quality’ of wine. The fact that our wines made with AWRI2965, with a concentration of TyrOH exceeding 120 mg L^−1^, were not rated differently to the control wine in palate attributes such as ‘astringency’, ‘bitterness’ or ‘sourness’, suggest a limited effect of this higher alcohol on white wine in-mouth sensory properties.

Over time in-bottle, the sensory differences among the wines produced by each yeast became larger. Although the magnitude of the strain effect on ‘floral/rose aroma’ was similar across the two time points (Tables S4 and S6), an aroma quality shift was indicated, with the more general ‘floral aroma’ attribute of the first study replaced with a specific odour quality of ‘rose aroma’ in the second study. The appearance of ‘rose aroma’ may result from a decrease in the concentration of other compounds such as monoterpenes and acetate esters through acid hydrolysis, which likely also contributed similar floral and fruit nuances to the young wines, and that could have overshadowed the specific ‘rose aroma’. Over time in-bottle we observed a pronounced decrease, between 45% to 70% depending on the strain, in the concentration of some acetate esters (Tables S8 and S9). The concentration of 2-PEA also decreased with time (between 40–55%) in wines made with the yeast variants; however, the concentration of this compound remained at levels that would likely transmit the ‘rose aroma’ character to the wines even after 15 months of aging. In wines made using the parent strain, 2-PEA concentration decreased to a level unlikely to strongly direct ‘rose aroma’. Practically, this finding may provide a method to extend the shelf-life of floral wine styles.

Another aspect related to the effect of aging is the formation of aldehydes from oxidation of the analogous higher alcohols. These aldehydes can have a much lower sensory threshold than their corresponding higher alcohols; for example, the sensory threshold for methional is about 1000-times lower than that of methionol [41,42]. Therefore, even limited oxidation can affect wine aroma substantially. In particular, methional has been linked with the ‘cooked vegetable’ off-flavour observed in oxidised wine [42]. Even though a general increase in the concentration of methional with storage time was observed, wines made with the variants AWRI2965 and AWRI2940 still showed lower concentrations of this compound than those made with the parent strain, and had lower scores in ‘cooked vegetable/potato’ aroma. Interestingly, although AWRI2940 produced an elevated concentration of methionol (also with a ‘cooked vegetable/potato’ aroma), this did not seem to contribute to the ‘vegetal aroma’ rated in wines made with this strain. The lack of a correlation between methionol and ‘vegetal aroma’ could be due to the masking effect of high concentrations of 2-PE present in the wines made with AWRI2940.

The production of methionol and other negative VSCs has been shown to be affected by the branch of the Ehrlich pathway responsible for the production of aromatic higher alcohols [6]. Recently, it has been shown that the deletion of *ARO8* in a wine strain, encoding for the aromatic transaminase Aro8p, decreased methionol formation after fermentation of a synthetic grape must [6]. The decrease in methionol formation in *ARO8* mutants indicates that by blocking the aromatic Ehrlich pathway, the catabolism of methionine might also be impaired.

Conversely, a highly active Ehrlich pathway may result in higher catabolism of methionine to methionol. The aromatic transaminase Aro9p, as well as the decarboxylase Aro10p, have been shown to be involved in the catabolism of both aromatic amino acids and methionine [5,7,8], and the expression of both *ARO9* and *ARO10* genes are highly induced by aromatic amino acids [43] and the end product TOL [44]. In this work, upregulation of the first step of the Ehrlich pathway was confirmed in two variants (AWRI2940 and 2965) by using an *ARO9*-promoter-BFP reporter gene (Figure S4). Therefore, we can hypothesise that the overproduction of aromatic amino acids and/or TOL in the different variants used in our study might lead to an activation of these two critical steps in the Ehrlich pathway and an increase in the catabolism of methionine to form methionol. The Ehrlich pathway-mediated catabolism of methionine, in turn, may limit the amount of methionine available for the formation of MeSH, methional, and MeSAc by the competing demethiolation pathway and/or by non-enzymatic reactions.

The idea that there is competition for methionine between the Erhlich and demethiolation pathways is supported by the VSC profile observed in the pilot-scale wines made with the three homozygous Tyr1p mutants, particularly with the most active strain AWRI2940. Again, AWRI2965, which harbours a mutation (Aro4p) earlier in the amino acid biosynthetic pathway, behaved somewhat differently than the Tyr1p variants. Higher concentrations of methionol were not observed in the wines produced using AWRI2965 but decreased concentrations of other undesirable VSCs such as MeSH, MeSAc or methional were observed. Interestingly, AWRI2965 also produced slightly higher concentrations of DMS in these conditions. Even though DMS is also associated with reductive off-odours and ‘vegetal’ aromas, at low concentrations (about 25 µg L^−1^) it can be described as contributing ‘blackcurrant’ and ‘red fruit’ aromas, and it is considered to enhance the bouquet of some wine styles [45,46].

## 4. Materials and Methods

### 4.1. Microorganisms and Culture Conditions

The commercial diploid wine strain AWRI796, and its five variants AWRI2936, AWRI2940, AWRI2965, AWRI4124, and AWRI2969, were obtained from The Australian Wine Research Institute (AWRI) culture collection (Table S1). These variants had been isolated previously using toxic analogues of the amino acid phenylalanine, as described in [23]. Yeast cultures were maintained on solid YPD agar plates (2% glucose, 2% peptone, 1% yeast extract, and 2% agar).

### 4.2. Laboratory-Scale Fermentation in a Synthetic Grape Medium

Laboratory-scale fermentations were performed in triplicate in a synthetic grape medium (SGM) [47], with a concentration of 6 mg L^−1^ of methionine. SGM was filtered through 0.22 µm Stericup filters (Millipore). Yeast starter cultures were prepared by growing cells aerobically in YPD medium for 24 h to stationary phase at 22 °C. Then, 1 × 10^6^ cells mL^−1^ were inoculated into 50% diluted SGM medium and grown for another 48 h at 22 °C. The acclimatised cells were inoculated into 100 mL of SGM at a density of 1 × 10^6^ cells mL^−1^. Fermentations were conducted at 17 °C in 100 mL glass bottles (Schott Duran), fitted with stir bars and stirred at 200 rpm using a magnetic stirrer. The lids of the bottles were fitted with selective H_2_S detector tubes (Komyo, Kitagawa, Japan) to measure the release of H_2_S during fermentation. Fermentation progress was followed by CO_2_ weight loss, measured every 24 h. After fermentation, the wines were cold settled at 4 °C for 5 days and sampled for different volatile and non-volatile compound analyses.

### 4.3. Pilot-Scale Winemaking

The pilot-scale winemaking trial with Chardonnay juice was performed by the Wine Innovation Cluster (WIC) winemaking services, according to a standardised white winemaking protocol. Hand-harvested Chardonnay grapes from the McLaren Vale region (South Australia, Australia) were used. The basic chemical parameters of the Chardonnay juice were: 12.7 °Baumé, yeast assimilable nitrogen 217 mg L^−1^, and pH 3.29. A concentration of 25 mg L^−1^ of SO_2_ was added to the grape must at the crusher. Yeast strains were grown for 48 h in filter-sterilised neutral grape concentrate (Tarac Technologies, Nuriootpa, Australia), which had been previously diluted to ~6 °Baume and pH adjusted to 3.5. Cells were inoculated at a density of approximately 2 × 10^6^ cells mL^−1^ in 19 L of the Chardonnay juice, and fermentation was conducted at 15 °C in 20 L stainless steel kegs in triplicate. When °Baumé was below 3, wines were moved to 20 °C. Irrespective of the starter culture used, wines got stuck around 1 °Baumé (day 16–18 of fermentation). Ferments were then rescued at day 26 by the addition of the commercial yeast Lalvin EC 1118 (Lallemand, Adelaide, SA, Australia). Once alcoholic fermentation had finished (day 31), wines were sulfured with 80 mg L^−1^ of SO_2_, and cold-stabilised for approximately 2 months at 0 °C. Before bottling, SO_2_ concentration was adjusted to between 35–40 mg L^−1^ of free SO_2_. Screw-cap sealed bottled wines (375 mL) were stored in the dark at a constant temperature of 15 °C.

### 4.4. Targeted Analyses of Volatile Compounds

Targeted analyses of fermentation-derived compounds (higher alcohols, acids, and esters) were performed by Metabolomics Australia (Adelaide) by GC-MS using a stable isotope dilution assay [48] at the end of fermentation, as well as 3 and 15 months post-bottling.

Analysis of monoterpenoids (linalool, *cis*-rose oxide, α-terpineol, nerol, geraniol) and C_13_-norisoprenoids (β-damascenone and β-ionone) was performed at 3 months post-bottling by GC-MS on an Agilent 6890 gas chromatograph equipped with a Gerstel MPS2 autosampler and coupled to an Agilent 5973N mass selective detector. Sample preparation was as follows: 10 mL of wine was transferred into a 20 mL crimp-cap, headspace-SPME vial (Grace Davison) with 3 g of NaCl followed by 50 µL of a combined d_4_-β-damascenone, d_3_-α-ionone and d_3_-β-ionone internal standard solution. Instrument control was performed with Agilent G1701EA Revision E.02.02 ChemStation software. The gas chromatograph was fitted with an Agilent DB-5ms 30 m × 0.25 mm × 0.5 um. Helium (Ultra High Purity) was used as the carrier gas with linear velocity 46 cm/s, flow rate 1.6 mL/min in constant flow mode. The oven temperature was started at 40 °C, held at this temperature for 2 min, then increased to 190 °C at 8 °C/min and held at this temperature for 5.25 min. The vial and its contents were heated to 60 °C for 10 min in the heater/agitator with the agitator on for 5 s and off for 2 s at 500 r.p.m. A Supelco grey 2 cm SPME fibre was exposed to the sample during this heating time through the septum. The fibre was then injected into a split/splitless inlet in splitless mode. The analytes were desorbed into a Supelco 0.75 mm ID sleeveless SPME liner for 10 min, which was held at 200 °C. The purge flow to the split vent was 50 mL/min at 2.1 min with the septum purge flow turned off. The mass spectrometer quadrupole temperature was set at 150 °C, the source was set at 230 °C and the transfer line was held at 250 °C. EMV Mode was set to Gain Factor = 1.00 and spectra were recorded in SIM mode.

### 4.5. Analysis of Volatile Sulfur Compounds (VSCs) and Aldehydes

The VSCs H_2_S, MeSH, DMS, diethyl sulfide, dimethyl disulfide, diethyl disulfide, ethanethiol, carbon disulfide, MeSAc, and ethyl thioacetate were quantified using an Agilent 355 sulfur chemiluminescence detector coupled to an Agilent 6890A gas chromatograph (Forest Hill, Melbourne, VIC, Australia), as described previously [49]. Reference standards of the different compounds were of the highest purity as supplied by Sigma-Aldrich (Castle Hill, Sydney, NSW, Australia) and Lancaster Synthesis (Jomar Bioscience, Adelaide, SA, Australia). Sodium hydrosulfide hydrate and sodium thiomethoxide were used as standards for H_2_S and MeSH, respectively. Ethylmethyl sulfide and propyl thioacetate were used as internal standards. Analytes were identified by comparison of their retention times with those of the corresponding pure reference compounds.

Analysis of the sulfur-containing compounds methionol and methional, as well as 2-methylpropanal, 3-methylbutanal, furfural, 5-methylfurfural, benzaldehyde, and 2-phenylacetadehyde was performed by GC-MS/MS, as described in [50]. Aldehydes were determined after derivatisation directly in the wine with *O*-(2,3,4,5,6-pentafluorobenzyl)hydroxylamine hydrochloride (Sigma-Aldrich). Reference standards for these compounds of the highest purity were purchased from Sigma-Aldrich. Isotopically labelled analogues for furfural, methionol, methional, benzaldehyde, and 2-phenylacetaldehyde were used as internal standards for accurate quantification of these compounds. For the quantitation of 2-methylpropanal and 3-methylbutanal, *d*_5_-benzaldehyde was used as an internal standard. Similarly, *d*_4_-furfural was used for the determination of 5-methylfurfural. With the exception of *d*_4_-furfural (CDN Isotopes, Sydney, NSW, Australia), the synthesis of the other isotopically labelled standards was carried out in-house as described in [50].

VSCs and aldehydes were analysed 3 and 15 months post-bottling.

### 4.6. Analysis of Principal Non-Volatile Compounds

The concentrations of sugars, ethanol, glycerol, and organic acids (acetic, malic, and succinic) were measured by HPLC using a Bio-Rad HPX-87H column, as described previously [51]. Reference standards of the highest purity were obtained from Sigma-Aldrich.

TyrOH, TOL, and TOL-SO_3_H were analysed on an Agilent 1200SL HPLC using a Phenomenex Kinetex PFP column (2.6 µm particle size, 2.1 mm × 150 mm) at different time points (end of alcoholic fermentation, and then 3, 6, 12, and 15 months post-bottling). The injection volume was 5 µL. The column was eluted at 45 °C with a gradient of 0.1% formic acid in Milli-Q water (A) and 0.1% formic acid in acetonitrile (B) at a flow rate of 0.4 mL min^−1^. The gradient was as follows: an initial isocratic hold (0% B) for 8 min, then gradient to 5% B over 32 min, gradient to 25% B over 9 min, then gradient to 80% B over 3 min, held isocratically at 80% B for 3 min, and dropped to 0% B and held for another 15 min. Absorbance at 280 nm was monitored with an Agilent 1260 Series G7117C DAD, while fluorescence was monitored at excitation and emission wavelengths of 280 and 350 nm, respectively, with an Agilent 1260 Series G7121B FLD. Quantification of TOL and TyrOH was performed using the absorbance detector, while TOL-SO_3_H was quantified using the fluorescence detector. Reference standards for TyrOH and TOL were obtained from Sigma-Aldrich. TOL-SO_3_H was synthesised as previously described by Arapitsas, Guella and Mattivi [16] with modifications. Briefly, TOL solution (2.5 g in 200 mL EtOH) was slowly poured into a potassium metabisulfite solution (5 g in 500 mL H_2_O) with stirring and reacted at room temperature for 48 h. The reaction product was dried under a vacuum (30 °C) and dissolved in H_2_O. The product was purified using preparative HPLC with a Dionex UltiMate 3000 system, a C_18_ Synergi Hydro RP column (250 × 21.2 mm, 4 µm pore size, Phenomenex, Lane Cove, Australia), and solvent system of 100% H_2_O (A) and 100% acetonitrile (B). Gradient: 0–10 min 0% B, 10–25 min 50% B, 25–35 min 100% B, 8 mL/min flow rate. The structure of TOL-SO_3_H was confirmed using HRMS and NMR (400 MHz, Bruker, Germany) with samples in D_2_O at 300 K. Results were processed using Topspin software. M-H mass (m/z): 240.0325; Chemical shifts for ^1^H-NMR (400 MHz, D_2_O) and ^13^C-NMR (D_2_O) concur with those previously reported [16].

### 4.7. Sensory Evaluation

Quantitative descriptive analysis (QDA) sensory studies [52] were conducted on the Chardonnay wines at two time points (3 months and 15 months post-bottling); however, wine produced with strain AWRI2969 was not included in the second study. Two panels of 10 judges with average ages of 48 (SD = 9.2, nine females, one male) and 50 (SD = 6.8, eight females, two males) years, respectively, were convened for each study. All panellists were part of the external AWRI trained descriptive analysis panel and had extensive experience in wine QDA. For both evaluations, assessors attended three two-hour training sessions to determine appropriate descriptors for rating in the formal sessions. For the 15-month evaluation, attributes used from the 3-month study were presented for consideration. No other information about the samples was given to the assessors at the second time point. All the wines from the study were progressively used during training sessions and appropriate attributes and definitions describing the appearance, aroma, and palate were agreed upon by judges in a consensus-based approach. Sensory standards for these descriptive attributes were presented, discussed, and recipes refined to represent attributes rated for the wines closely. These standards were available during all subsequent sessions and panellists revisited them at the beginning of each formal assessment session. The attributes rated, definitions, and standard recipes can be found in Tables S8 and S9, while the chemical composition of the wines is summarised in Tables S10 and S11. In both studies, samples were presented to panellists in 30 mL aliquots in 3-digit-coded, covered, ISO standard wine glasses at 22–24 °C, in isolated booths under daylight-type lighting, with randomised presentation order (modified Williams Latin Square). In the 3-month evaluation, wines were presented to the panel in duplicate while in the 15-month evaluation, the wines were presented in triplicate. Assessors were forced to have a 60 s rest between samples and were encouraged to rinse with water, and a minimum 10 min rest between sets of three samples. During the 10 min break, assessors were requested to leave the booths. For the 3-month evaluation, 12 samples were presented per day while for the 15-month evaluation, 15 samples were assessed per day. All samples were expectorated. Compusense Cloud sensory evaluation software (Compusense Inc., Guelph, Canada) was used on both occasions to generate presentation replicate designs and collect sensory data. The intensity of each attribute was rated using an unstructured 15 cm line scale (numericised 0 to 10), with indented anchor points of ‘low’ and ‘high’ placed at 10% and 90%, respectively. Panel performance was assessed using Compusense software and R with the SensomineR (sensominer.free.fr/) and FactomineR (factominer.free.fr/) packages.

### 4.8. Statistical Analysis

Minitab 19 (Minitab Inc., Sydney, NSW, Australia) was used for statistical analysis of the compositional data which were analysed by one-way analysis of variance (ANOVA). Multiple comparisons of the analyte concentration with respect to treatment were undertaken using Tukey’s honestly significant difference (HSD) test (alpha = 0.05), and *p* values were determined by a two-tailed Student’s t test. For the sensory data, ANOVA was carried out using Minitab 19. The effects of the yeast strain treatment, judge, judge by strain, ferment replicate nested into strain, judge by ferment replicate nested into strain, presentation replicate nested into strain, and ferment replicate were assessed, treating judge as a random effect. Following ANOVA, a protected HSD value was calculated using the mean square term of the judge × strain interaction at a 95% confidence level for attributes with a significant (*p* < 0.05) treatment effect. To explore the relationship between wine chemical composition and sensory attributes, PLS-R was conducted for each wine replicate, as described in [53] with some modifications. Sensory attribute responses (Ys) were included in the models if some statistical evidence (*p* < 0.10) signalled a treatment effect or a high F-ratio was found indicating potential treatment effects which may have been overshadowed by judge, fermentation, or presentation replicate variation.

## 5. Conclusions

This study confirmed that the higher alcohol overproduction phenotype of five variants derived from the commercial wine strain AWRI796 is maintained in pilot-scale white winemaking conditions. This overproduction was associated with meaningful changes in wine sensory profiles, especially after some period of bottle storage. The effect of these strains on wine chemical composition was not just limited to the overproduction of 2-PE but also to an increase in the concentration of the higher alcohols TyrOH and/or TOL and to the formation of VSCs. Associations between these compounds and ‘sweet’, ‘sour’ and ‘bitter’ tastes, and ‘cooked vegetable/potato aroma’, were identified. These results highlight the intricate connections between the metabolism of aromatic amino acids and the sulfur-containing amino acid methionine during fermentation, ultimately influencing wine flavour.

The various yeast strains isolated in this study provide novel tools for winemakers to adjust and preserve wine style. In particular, AWRI2965 has excellent potential as a white wine winemaking yeast, imparting accentuated and lasting rose/floral aromas to wines.

More research will be needed to understand the compositional drivers of bitterness. In particular, the role of higher alcohols derived from the metabolism of aromatic amino acids TyrOH, TOL, and its sulfonated derivative TOL-SO_3_H, needs to be elucidated along with the physico-chemical conditions such as pH, temperature, storage time, and SO_2_ concentration, which might influence the equilibrium between these compounds in the finished wine.

## Figures and Tables

**Table 1:** Mean scores for a subset of appearance, aroma, and palate attributes of the yeast strains after 3 months in-bottle.

Yeast	Appearance	Aroma						Palate					
	Yellow Colour	Stone Fruit	Pungent	Floral	Grassy	Flint	Cooked Veg/Potato	Sweetness	Bitterness	Astringency	Stone Fruit F	Grassy F	Flint F
AWRI796	3.85	3.56	4.87	4.82	2.96	1.61	1.62	1.95	4.90	3.43	3.82	3.31	1.69
AWRI2936	4.06	3.53	4.99	4.61	2.38	1.61	1.35	1.87	4.92	3.59	3.82	3.12	1.66
AWRI2940	4.47	3.70	5.05	5.28	2.67	1.31	1.08	1.46	5.19	3.57	3.47	3.53	1.48
AWRI2965	4.03	3.66	5.02	4.68	2.92	1.90	1.28	1.42	5.26	3.61	3.32	3.33	2.02
AWRI2969	4.03	3.81	4.78	4.68	2.78	1.57	1.32	1.48	5.21	3.59	3.31	3.54	1.55
AWRI4124	4.09	3.78	4.85	4.42	2.72	1.40	1.86	1.69	5.21	3.64	3.76	3.36	1.75
HSD	0.37	ns	ns	0.79	ns	ns	ns	ns	ns	ns	ns	0.41	ns

F: Flavour. HSD (*p* = 0.05) values included for the significant attributes (*p* < 0.05), ns: not significantly different.

**Table 2:** Mean scores for a subset of appearance, aroma, and palate attributes of the yeast strains after 15 months in-bottle.

Yeast	Appearance	Aroma						Palate					
	Yellow Colour	Stone Fruit	Pungent	Rose	Grassy	Flint	Cooked Veg/Potato	Sweetness	Bitterness	Astringency	Stone Fruit F	Sourness	Rose F
AWRI796	4.05	3.24	4.41	3.72	2.31	1.43	2.38	1.44	3.44	2.80	3.31	5.04	2.07
AWRI2936	4.13	3.45	4.56	3.94	2.22	1.45	1.66	1.64	3.42	2.64	3.66	5.09	2.19
AWRI2940	4.79	3.28	4.37	4.55	2.21	1.16	1.06	0.78	3.78	2.86	2.96	5.46	2.77
AWRI2965	4.19	3.51	4.20	4.03	2.11	1.42	1.14	1.46	3.47	2.83	3.42	5.10	2.24
AWRI4124	4.40	3.19	4.47	4.06	2.11	1.61	2.36	1.37	3.47	2.84	3.29	5.29	2.31
HSD	0.27	ns	0.33	0.75	ns	ns	0.72	0.49	ns	ns	0.61	0.29	ns

F: Flavour. HSD (*p* = 0.05) values included for the significant attributes (*p* < 0.05), ns: not significantly different.

## Data Availability

All data has been made available through the manuscript itself or via supplemental materials.

## Supplementary Materials

The following are available online, Table S1. Strains used in this study; Table S2. Higher alcohols and esters produced following alcoholic fermentation of Chardonnay; Table S3. Basic wine composition at bottling of Chardonnay wines; Table S4. F-ratios, probability values, degrees of freedom, and mean square error from the analysis of variance conducted following sensory analysis of wines after 3 months in-bottle; Table S5. Mean scores and Tukey’s HSD values for sensory attributes after 3 months in-bottle; Table S6. F-ratios, probability values, degrees of freedom, and mean square error from the analysis of variance conducted following sensory analysis of wines after 15 months in-bottle; Table S7. Mean scores and Tukey’s HSD values for sensory attributes after 15 months in-bottle; Table S8. Sensory attributes, definitions and composition of reference standards after 3 months in-bottle; Table S9. Sensory attributes, definitions and composition of reference standards after 15 months in-bottle; Table S10. Composition of the wines after 3 months in-bottle; Table S11. Composition of the wines after 15 months in-bottle. Figure S1. Relationship between 2-PE and 2-PEA production at the end of alcoholic fermentation in the Chardonnay wines; Figure S2. Relationship between concentrations of TOL at the end of alcoholic fermentation and its maximum percentage of conversion into TOL-SO_3_H in the Chardonnay wines; Figure S3. Relationship between the decrease in free and total SO_2_ concentrations and TOL-SO_3_H formation in the Chardonnay wines at different time points during ageing; Figure S4. Quantitative assay of the activation of the *ARO9* promoter by using a reporter system.

## Sample Availability

Samples are available from the authors for a limited time. Wine will not be stored indefinitely.
