# Supplementary material for: Aromatic Higher Alcohols in Wine: Implication on Aroma and Palate Attributes during Chardonnay Aging
Source: Molecules. 2021 Aug 17;26(16):4979. doi: 10.3390/molecules26164979 (PMC8400268; doi:10.3390/molecules26164979)

**Table S1.** Strains used in this study.

| Yeast strain (AWRI number) | Selection[a] | Causative mutation[b] |
|---|---|---|
| AWRI796 | | |
| AWRI2936 | PFP (spontaneous) | Tyr1p C108Y[†] |
| AWRI2940 | PFP (spontaneous) | Tyr1p M197I |
| AWRI4124 | PFP (mutagenized) | Tyr1p A200T |
| AWRI2965 | OFP (spontaneous) | Aro4p Q166R |
| AWRI2969 | OFP (mutagenized) | Tyr1p S398L |

[a]Strategy used to select strains from parent AWRI796. Untreated (spontaneous) or chemically mutagenized cells were selected in solid media containing either *p*-fluoro-DL-phenylalanine (PFP) or *o*-fluoro-DL-phenylalanine (OFP). [b]Mutations in the amino acid sequence of proteins involved in aromatic amino acid metabolism ([†]mutation in heterozygosity).

**Table S2.** Higher alcohols and esters produced following alcoholic fermentation of Chardonnay using strains AWRI796 (parent) and five variants carrying mutations in Aro4p (AWRI2965) or Tyr1p (AWRI2936, 2940, 4124, and 2969). Concentrations of the different metabolites are shown in mg $L^{-1}$, unless otherwise indicated.

| Metabolite | AWRI796 | AWRI2936 | AWRI2940 | AWRI4124 | AWRI2965 | AWRI2969 | P-value | F-value |
|---|---|---|---|---|---|---|---|---|
| Ethyl acetate | 23.2 ± 1.5 | 25.0 ± 1.0 | 27.3 ± 0.88 | 27.6 ± 0.86 | 25.3 ± 1.0 | 24.2 ± 1.5 | 0.040 | 6.6 |
| 2-methylpropyl acetate (Val) ($\mu$g $L^{-1}$) | 89 ± 29$^{b}$ | 120 ± 34$^{ab}$ | 176 ± 3.8$^{a}$ | 112 ± 29$^{ab}$ | 85 ± 24$^{b}$ | 97 ± 17$^{ab}$ | 0.033 | 3.6 |
| 2-methylbutyl acetate (Ile) ($\mu$g $L^{-1}$) | 108 ± 5.2$^{d}$ | 160 ± 3.7$^{c}$ | 209 ± 10$^{a}$ | 195 ± 5.1$^{a}$ | 147 ± 3.6$^{c}$ | 178 ± 4.3$^{b}$ | <0.001 | 118 |
| 3-methylbutyl acetate (Leu) ($\mu$g $L^{-1}$) | 2649 ± 102$^{c}$ | 2848 ± 87$^{c}$ | 4064 ± 213$^{a}$ | 3552 ± 70$^{b}$ | 2895 ± 37$^{c}$ | 3490 ± 110$^{b}$ | <0.001 | 64 |
| 2-PEA (Phe) | 0.28 ± 0.03$^{f}$ | 1.48 ± 0.05$^{e}$ | 9.88 ± 0.30$^{a}$ | 4.08 ± 0.07$^{c}$ | 3.55 ± 0.03$^{d}$ | 4.93 ± 0.02$^{b}$ | <0.001 | 1973 |
| 2-methylpropanol (Val) | 28.5 ± 5.0$^{ab}$ | 27.8 ± 0.6$^{ab}$ | 36.5 ± 3.0$^{a}$ | 32.8 ± 3.8$^{ab}$ | 22.3 ± 1.6$^{b}$ | 25.9 ± 2.1$^{ab}$ | 0.074 | 2.7 |
| 2-methylbutanol (Ile) | 19.7 ± 1.5 | 23.3 ± 3.6 | 25.7 ± 2.0 | 25.3 ± 0.95 | 22.1 ± 4.9 | 22.8 ± 1.2 | 0.159 | 1.9 |
| 3-methylbutanol (Leu) | 114.0 ± 9.1 | 110.7 ± 10.1 | 122.5 ± 7.4 | 121.2 ± 2.0 | 108.8 ± 20.1 | 119.0 ± 4.8 | 0.532 | 0.9 |
| 2-PE (Phe) | 19.8 ± 2.9$^{e}$ | 53.9 ± 2.4$^{d}$ | 301.5 ± 12.7$^{a}$ | 138.9 ± 0.70$^{c}$ | 147.4 ± 2.3$^{c}$ | 164.8 ± 1.0$^{b}$ | <0.001 | 966 |
| TyrOH (Tyr) | 13.6 ± 1.8$^{b}$ | 10.1 ± 0.1$^{c}$ | 7.1 ± 0.2$^{d}$ | 9.1 ± 0.2$^{cd}$ | 127 ± 1.6$^{a}$ | 7.5 ± 0.1$^{cd}$ | <0.001 | 7205 |
| TOL (Trp) | 0.89 ± 0.02$^{e}$ | 2.66 ± 0.1$^{d}$ | 52.1 ± 0.5$^{a}$ | 6.3 ± 0.3$^{c}$ | 7.8 ± 0.4$^{b}$ | 5.7 ± 0.2$^{c}$ | <0.001 | 11240 |
| TOL-$SO_3H$ | <LOQ | <LOQ | 0.26 ± 0.13 | <LOQ | <LOQ | <LOQ | - | - |
| Free $SO_2$ | <3 | <3 | <3 | <3 | <3 | <3 | - | - |
| Total $SO_2$ | 20 ± 1$^{ab}$ | 22 ± 1$^{a}$ | 22 ± 1$^{ab}$ | 22 ± 1$^{a}$ | 23 ± 2$^{a}$ | 18 ± 2$^{b}$ | 0.009 | 5.2 |

Results are the average ± standard deviation of three independent replicates. In parenthesis, amino acid from which the corresponding metabolite is derived from in the Ehrlich pathway. Means with the same superscript letter are not significantly different from each other (Tukey's test, $P < 0.05$).

**Table S3**. Basic wine composition at bottling of the Chardonnay wines made using the yeast AWRI796 (parent), and five variants carrying mutations in Aro4p (AWRI2965) or Tyr1p (AWRI2936, 2940, 4124, and 2969).

| strain | Ethanol (% v/v) | Sugars (g $L^{-1}$) | pH | Titratable acidity pH 8.2 | Malic (g $L^{-1}$) | Acetic acid (g $L^{-1}$) | Glycerol (g $L^{-1}$) | Free $SO_2$ (mg $L^{-1}$) | Total $SO_2$ (mg $L^{-1}$) | Molar ratio (Free $SO_2$ / TOL) |
|---|---|---|---|---|---|---|---|---|---|---|
| AWRI796 | 13.3 ± 0.1 | 0.6 ± 0.3 | 3.42 ± 0.0 | 5.7 ± 0.2 | 2.1 ± 0.1 | 0.39 ± 0.02 | 5.9 ± 0.1 | 42 ± 0.6 | 109 ± 1.5 | 132 |
| AWRI2936 | 13.5 ± 0.1 | 0.7 ± 0.1 | 3.42 ± 0.0 | 5.6 ± 0.1 | 2.1 ± 0.0 | 0.35 ± 0.01 | 5.7 ± 0.1 | 40 ± 1.2 | 112 ± 1.2 | 39 |
| AWRI2940 | 13.4 ± 0.0 | 1.1 ± 0.1 | 3.40 ± 0.01 | 5.7 ± 0.0 | 2.2 ± 0.1 | 0.40 ± 0.02 | 6.1 ± 0.1 | 38 ± 0.6 | 109 ± 0.6 | 1.8 |
| AWRI4124 | 13.4 ± 0.0 | 0.7 ± 0. 1 | 3.43 ± 0.01 | 5.7 ± 0.0 | 2.0 ± 0.1 | 0.43 ± 0.01 | 6.4 ± 0.1 | 36 ± 0.6 | 109 ± 0.6 | 15 |
| AWRI2965 | 13.4 ± 0.1 | 1.1 ± 0.1 | 3.43 ± 0.01 | 5.6 ± 0.0 | 2.0 ± 0.1 | 0.46 ± 0.01 | 5.9 ± 0.1 | 40 ± 1.0 | 113 ± 0.6 | 13 |
| AWRI2969 | 13.4 ± 0.1 | 1.0 ± 0.2 | 3.43 ± 0.0 | 5.6 ± 0.0 | 2.1 ± 0.1 | 0.45 ± 0.01 | 5.9 ± 0.2 | 37 ± 0.6 | 105 ± 1.5 | 17 |

Results are the average ± standard deviation of three independent replicates.

**Table S4.** F-ratios, probability values†, degrees of freedom (df) and mean square error (MSE) from the analysis of variance of the sensory attribute data after 3 months in bottle.

| Attribute | Strain | FRep (Strain) | J*Strain | J*FRep (Strain) | PRep (Strain, FRep) | MSE |
|---|---|---|---|---|---|---|
| Yellow colour intensity | 5.71*** | 1.75 | 1.54* | 0.95 | 1.27 | 0.305 |
| Tropical fruit A | 1.42 | 0.99 | 0.72 | 0.81 | 0.75 | 1.190 |
| Passionfruit A | 0.10 | 1.69 | 1.43 | 0.72 | 1.33 | 2.061 |
| Citrus A | 0.74 | 0.66 | 1.26 | 0.88 | 1.06 | 1.338 |
| Banana confection A | 0.69 | 0.46 | 0.63 | 1.44* | 1.30 | 1.954 |
| Stone fruit A | 0.57 | 0.99 | 0.77 | 0.95 | 0.59 | 1.908 |
| Floral A | 2.49* | 1.91* | 1.27 | 0.76 | 0.52 | 2.108 |
| Grassy A | 1.92ǂ | 1.47 | 1.04 | 1.16 | 0.72 | 1.126 |
| Yeasty A | 0.37 | 0.75 | 1.20 | 0.74 | 0.96 | 1.622 |
| Flint A | 1.08 | 1.94* | 1.14 | 1.10 | 1.84* | 1.855 |
| Honey A | 0.52 | 0.88 | 1.53* | 1.01 | 0.71 | 1.699 |
| Cooked veg/potato A | 1.96ǂ | 0.54 | 1.49* | 0.76 | 0.84 | 2.099 |
| Pungent A | 0.84 | 0.74 | 1.34 | 1.09 | 2.02* | 0.573 |
| Sweetness | 2.34ǂ | 1.27 | 1.21 | 1.02 | 1.48 | 1.095 |
| Sourness | 0.48 | 0.77 | 1.25 | 1.01 | 0.80 | 0.517 |
| Bitterness | 1.74ǂ | 1.59 | 0.85 | 0.88 | 1.23 | 1.207 |
| Astringency | 0.67 | 1.02 | 0.68 | 1.36* | 1.26 | 0.534 |
| Viscosity | 1.09 | 0.25 | 0.94 | 1.05 | 1.26 | 0.509 |
| Hotness | 1.24 | 0.43 | 0.71 | 0.73 | 0.99 | 1.175 |
| Tropical fruit F | 0.96 | 1.20 | 1.26 | 0.62 | 0.71 | 1.177 |
| Citrus F | 0.34 | 1.29 | 2.19* | 0.47 | 1.14 | 1.043 |
| Stone fruit F | 2.24ǂ | 0.57 | 0.86 | 1.19 | 0.93 | 1.549 |
| Banana confection F | 0.41 | 0.75 | 0.92 | 0.87 | 0.84 | 1.780 |
| Grassy F | 2.63* | 1.32 | 0.74 | 0.93 | 1.04 | 0.786 |
| Flint F | 1.98ǂ | 1.11 | 0.77 | 0.79 | 0.64 | 1.783 |
| df | 5 | 12 | 45 | 108 | 18 | 162 |

A: aroma, F: Flavour, AT: Aftertaste. †Statistical evidence levels are as follows: * $P \leq 0.05$; ** $P \leq 0.01$; *** $P \leq 0.001$; ǂ $P \leq 0.15$. df = degrees of freedom. Evidence was found for Judge effects for all attributes ($P<0.001$), Strain = Yeast strain treatment, FRep = Fermentation replicate, PRep = presentation replicate, J = Judge.

**Table S5.** Mean scores and Tukey's Honest Significant Difference (HSD) values for sensory attributes after 3 months in bottle.

| Yeast Strain | AWRI796 | AWRI2936 | AWRI2940 | AWRI2965 | AWRI2969 | AWRI4124 | HSD |
|---|---|---|---|---|---|---|---|
| Yellow colour intensity | 3.85 | 4.06 | 4.47 | 4.03 | 4.03 | 4.09 | 0.37 |
| Tropical fruit A | 5.36 | 5.13 | 5.41 | 5.32 | 5.40 | 5.14 | ne |
| Passionfruit A | 2.36 | 2.43 | 2.45 | 2.44 | 2.32 | 2.47 | ne |
| Citrus A | 4.16 | 3.83 | 4.10 | 4.16 | 4.01 | 3.91 | ne |
| Banana confection A | 3.33 | 3.44 | 3.37 | 3.05 | 3.37 | 3.44 | ne |
| Stone fruit A | 3.56 | 3.53 | 3.70 | 3.66 | 3.81 | 3.78 | ne |
| Floral A | 4.82 | 4.61 | 5.28 | 4.68 | 4.68 | 4.42 | 0.79 |
| Grassy A | 2.96 | 2.38 | 2.67 | 2.92 | 2.78 | 2.72 | ne |
| Yeasty A | 1.08 | 1.11 | 0.90 | 1.14 | 0.99 | 1.11 | ne |
| Flint A | 1.61 | 1.61 | 1.31 | 1.90 | 1.57 | 1.40 | ne |
| Honey A | 1.99 | 1.93 | 1.63 | 1.83 | 2.07 | 1.89 | ne |
| Cooked veg/potato A | 1.62 | 1.35 | 1.08 | 1.28 | 1.32 | 1.86 | ne |
| Pungent A | 4.87 | 4.99 | 5.05 | 5.02 | 4.78 | 4.85 | ne |
| Sweetness | 1.95 | 1.87 | 1.46 | 1.42 | 1.48 | 1.69 | ne |
| Sourness | 6.17 | 6.20 | 6.27 | 6.17 | 6.09 | 6.28 | ne |
| Bitterness | 4.90 | 4.92 | 5.19 | 5.26 | 5.21 | 5.21 | ne |
| Astringency | 3.43 | 3.59 | 3.57 | 3.61 | 3.59 | 3.64 | ne |
| Viscosity | 4.46 | 4.40 | 4.46 | 4.32 | 4.38 | 4.60 | ne |
| Hotness | 4.85 | 5.00 | 4.90 | 4.79 | 4.73 | 5.01 | ne |
| Tropical fruit F | 5.38 | 5.04 | 5.17 | 5.09 | 5.24 | 5.25 | ne |
| Citrus F | 5.12 | 5.08 | 5.29 | 5.21 | 5.20 | 5.11 | ne |
| Stone fruit F | 3.82 | 3.82 | 3.47 | 3.32 | 3.31 | 3.76 | ne |
| Banana confection F | 2.67 | 2.54 | 2.43 | 2.46 | 2.58 | 2.66 | ne |
| Grassy F | 3.31 | 3.12 | 3.53 | 3.33 | 3.54 | 3.36 | 0.41 |
| Flint F | 1.69 | 1.66 | 1.48 | 2.02 | 1.55 | 1.75 | ne |

HSD values calculated at 95% confidence level included for sensory attributes that showed statistical evidence ($P$<0.05) for differing across strains, ne: no statistical evidence found.

**Table S6.** F-ratios, probability values†, degrees of freedom (df) and mean square error (MSE) from the analysis of variance of the sensory attribute data after 15 months in bottle.

| Attribute | Strain | FRep (Strain) | J*Strain | J*FRep (Strain) | PRep (Strain, FRep) | MSE |
|---|---|---|---|---|---|---|
| Yellow colour intensity | 19.95*** | 3.5*** | 1.26 | 1.35* | 2.12*** | 0.235 |
| Pungent A | 2.75* | 1.01 | 1.46 | 0.68 | 0.75 | 0.592 |
| Stone fruit A | 1.81 | 1.89 | 0.62 | 1.49** | 1.21 | 1.022 |
| Rose A | 2.68* | 1.34 | 1.11 | 0.87 | 1.36 | 3.212 |
| Citrus A | 0.32 | 1.71 | 1.33 | 1.25 | 1.51* | 0.407 |
| Banana A | 1.82 | 0.94 | 0.77 | 0.85 | 1.04 | 2.219 |
| Grassy A | 0.93 | 0.91 | 0.41 | 1.25 | 1.37 | 0.695 |
| Flint A | 0.92 | 1.50 | 1.85** | 0.71 | 0.97 | 1.896 |
| Cooked veg/potato A | 12.75*** | 1.96* | 1.18 | 1.01 | 0.95 | 2.383 |
| Sourness | 5.69*** | 0.73 | 0.85 | 0.86 | 0.83 | 0.651 |
| Astringency | 1.00 | 0.83 | 1.52 | 0.65 | 1.30 | 0.723 |
| Bitterness | 1.45 | 1.93 | 1.12 | 1.09 | 0.83 | 1.130 |
| Viscosity | 0.22 | 0.46 | 1.42 | 0.70 | 0.97 | 0.620 |
| Hotness | 0.68 | 0.47 | 1.54 | 0.82 | 1.44 | 1.141 |
| Sweetness | 7.41*** | 3.32*** | 1.08 | 0.96 | 1.10 | 1.255 |
| Citrus F | 1.41 | 1.55 | 1.48 | 0.63 | 1.51* | 0.920 |
| Rose F | 2.49ǂ | 0.96 | 1.51 | 0.81 | 0.95 | 2.147 |
| Stone fruit F | 2.75* | 0.88 | 1.19 | 1.06 | 0.79 | 1.627 |
| df | 4 | 10 | 36 | 90 | 30 | 270 |

A: aroma, F: Flavour, AT: Aftertaste. †Statistical evidence levels are as follows: * $P \leq 0.05$; ** $P \leq 0.01$; *** $P \leq 0.001$; ǂ $P \leq 0.15$. df = degrees of freedom. Evidence was found for Judge effects for all attributes ($P<0.001$), Strain = Yeast strain treatment, FRep = Fermentation replicate, PRep = presentation replicate, J = Judge.

**Table S7.** Mean scores and Tukey's Honest Significant Difference values for sensory attributes after 15 months in bottle.

| Yeast Strain | AWRI796 | AWRI2936 | AWRI2940 | AWRI2965 | AWRI4124 | HSD |
|---|---|---|---|---|---|---|
| Yellow colour intensity | 4.05 | 4.13 | 4.79 | 4.19 | 4.40 | 0.27 |
| Pungent A | 4.41 | 4.56 | 4.37 | 4.20 | 4.47 | 0.33 |
| Stone fruit A | 3.24 | 3.45 | 3.28 | 3.51 | 3.19 | ne |
| Rose A | 3.72 | 3.94 | 4.55 | 4.03 | 4.06 | 0.75 |
| Citrus A | 2.68 | 2.62 | 2.63 | 2.56 | 2.57 | ne |
| Banana A | 2.40 | 2.54 | 2.55 | 2.72 | 2.27 | ne |
| Grassy A | 2.31 | 2.22 | 2.21 | 2.11 | 2.11 | ne |
| Flint A | 1.43 | 1.45 | 1.16 | 1.42 | 1.61 | ne |
| Cooked veg/potato A | 2.38 | 1.66 | 1.06 | 1.14 | 2.36 | 0.72 |
| Sourness | 5.04 | 5.09 | 5.46 | 5.10 | 5.29 | 0.29 |
| Astringency | 2.80 | 2.64 | 2.86 | 2.83 | 2.84 | ne |
| Bitterness | 3.44 | 3.42 | 3.78 | 3.47 | 3.47 | ne |
| Viscosity | 3.94 | 3.96 | 3.95 | 4.02 | 4.03 | ne |
| Hotness | 4.01 | 3.72 | 3.85 | 3.83 | 3.87 | ne |
| Sweetness | 1.44 | 1.64 | 0.78 | 1.46 | 1.37 | 0.49 |
| Citrus F | 4.32 | 4.18 | 4.46 | 4.19 | 4.34 | ne |
| Rose F | 2.07 | 2.19 | 2.77 | 2.24 | 2.31 | ne |
| Stone fruit F | 3.31 | 3.66 | 2.96 | 3.42 | 3.29 | 0.61 |

HSD values calculated at 95% confidence level included for sensory attributes that showed statistical evidence ($P<0.05$) for differing across strains, ne: no statistical evidence found.

**Table S8.** Sensory attributes, definitions and composition of reference standards after 3 months in bottle.

| Attribute | Definition/Synonyms | Standard |
|---|---|---|
| ***Appearance*** | | |
| Yellow colour | The degree of yellow colour intensity in the sample | |
| ***Aroma*** | | |
| Tropical fruit | Intensity of the aroma of tropical fruits; pineapple and melon | 10 g cubes and juice of tinned pineapple (Golden Circle), 10 g cubes and juice of rockmelon fruit cup (SPC Ardmona) |
| Passionfruit | Intensity of the aroma of passionfruit, including sweaty odour | 2 µL of 16.5 mL/L 3-mercaptohexyl acetate |
| Citrus | Intensity of the aroma of citrus fruits | 5 g of fresh lemon, 5 g of fresh lime and 5g of fresh grapefruit including zest |
| Banana confection | Intensity of the aroma of banana lolly | 5 g banana lolly (Black & Gold Brand) not in wine |
| Stone fruit | Intensity of the aroma of stone fruit including; apricot and peach | 10 g apricots and 10 g peaches, in juice (Goulburn Valley) |
| Floral | Intensity of the aroma of orange blossom, rose and laundry powder | 40 µL of 200 mg/L 2-phenylethanol |
| Grassy | Intensity of the aroma of freshly cut grass and hay | 1g of fresh cut grass not in wine. |
| Yeasty | Intensity of the aroma of baker's yeast | 1 g of baker's yeast and 1 g sugar in 30 mL 35 $^{o}$C water |
| Flint | Intensity of the aroma of flint and struck match and mineral | 20 µL of 1 mg/L benzyl mercaptan |
| Honey | Intensity of the aroma of honey | 2.5 g honey (Beechworth's) |
| Cooked veg/potato | Intensity of the aroma of cooked veg water and boiled potato | 20 µL of 3.3 mg/L methional |
| Pungent | Intensity of the aroma and effect of alcohol. | 4 mL of 95% food grade ethanol (Tarac Technologies) |
| ***Palate*** | | |
| Sweetness | Intensity of the perception of sweetness | 6 g/L table sugar in water |
| Sourness | Intensity of acid taste in the mouth including aftertaste. | 2 g/L L-(+)-tartaric acid (Chem-Supply) in water |
| Bitterness | The intensity of bitter taste perceived in the mouth, or after expectoration. | 15 mg/L quinine sulfate (Sigma Aldrich) in water |

| Attribute | Definition/Synonyms | Standard |
|---|---|---|
| Astringency | The drying and mouth-puckering sensation in the mouth. Low = coating teeth; Medium = mouth coating & drying; High = puckering, lasting astringency. | 0.43 g/L aluminium sulfate (Ajax fine Chem Supply Pty Ltd in water |
| Viscosity | The perception of the body, weight or thickness of the wine in the mouth. Low = watery, thin mouth feel. High = oily, thick mouth feel. | 1.5 g/L carboxymethylcellulose sodium salt (Sigma Aldrich) in water |
| Hotness | The intensity of alcohol hotness perceived in the mouth, after expectoration and the associated burning sensation. Low = warm; High = hot, burning. | 8% food grade alcohol (Tarac Technologies) in water |
| Tropical fruit | Intensity of the flavour of tropical fruits; pineapple and melon | |
| Citrus | Intensity of the flavour of citrus fruits | |
| Stone fruit | Intensity of the flavour of stone fruit including apricot and peach | |
| Banana confection | Intensity of the flavour of banana lolly | |
| Grassy | Intensity of the flavour of freshly cut grass and hay | |
| Flint | Intensity of the flavour of flint, struck match and mineral | |

All white wine standards were added to 30 mL of 2017 Yalumba premium selection bag-in-box unoaked Chardonnay unless otherwise noted.

**Table S9.** Sensory attributes, definitions and composition of reference standards after 15 months in bottle

| Attribute | Definition/Synonyms | Standard |
|---|---|---|
| ***Appearance*** | | |
| Yellow colour | The degree of yellow colour intensity in the sample | |
| ***Aroma*** | | |
| Pungent | Intensity of the aroma and effect of alcohol. | 4 mL of 95% food grade ethanol (Tarac Technologies) |
| Stone fruit | Intensity of the aroma of stone fruit including; apricot and peach | 5 g apricots and 5 g peaches, in juice (Goulburn Valley) |
| Rose | Intensity of the aroma of floral roses | 2 mL of rosewater (ANOUN Food Co.) |
| Citrus | Intensity of the aroma of citrus fruits | 5 g of fresh lemon, 5 g of fresh lime and 5g of fresh grapefruit including zest |
| Banana | Intensity of the aroma of banana lolly | 5 g banana lolly (Black & Gold Brand) not in wine |
| Grassy | Intensity of the aroma of freshly cut grass and hay | 1g of fresh cut grass not in wine. |
| Flint | Intensity of the aroma of flint and struck match and mineral | 20 μL of 1 mg/L benzyl mercaptan |
| Cooked veg/potato | Intensity of the aroma of cooked veg water and boiled potato | 20 μL of 3.3 mg/L methional |
| ***Palate*** | | |
| Sourness | Intensity of acid taste in the mouth including aftertaste. | 2 g/L L-(+)-tartaric acid (Chem-Supply) in water |
| Astringency | The drying and mouth-puckering sensation in the mouth. Low = coating teeth; Medium = mouth coating & drying; High = puckering, lasting astringency. | 0.43 g/L aluminium sulfate (Ajax fine Chem Supply Pty Ltd in water |
| Bitterness | The intensity of bitter taste perceived in the mouth, or after expectoration. | 15 mg/L quinine sulfate (Sigma Aldrich) in water |
| Viscosity | The perception of the body, weight or thickness of the wine in the mouth. Low = watery, thin mouth feel. High = oily, thick mouth feel. | 1.5 g/L carboxymethylcellulose sodium salt (Sigma Aldrich) in water |

| Attribute | Definition/Synonyms | Standard |
|---|---|---|
| Hotness | The intensity of alcohol hotness perceived in the mouth, after expectoration and the associated burning sensation. Low = warm; High = hot, burning. | 8% food grade alcohol (Tarac Technologies) in water |
| Sweetness | Intensity of the perception of sweetness | 6 g/L table sugar in water |
| Citrus | Intensity of the flavour of citrus fruits | |
| Rose | Intensity of the flavour of floral roses and Turkish delight confection | |
| Stone fruit | Intensity of the flavour of stone fruit including apricot and peach | |

All white wine standards were added to 30 mL of 2017 Yalumba premium selection bag-in-box unoaked Chardonnay unless otherwise noted.

**Table S10.** Composition of the wines after 3 months in bottle. Wines were produced using AWRI796 (parent strain) and five variants carrying mutations in Aro4p (AWRI2965) or Tyr1p (AWRI2936, 2940, 4124, and 2969). Concentrations of the different metabolites are shown in μg L$^{-1}$, unless otherwise indicated.

| Metabolite | Label | AWRI796 | AWRI2936 | AWRI2940 | AWRI4124 | AWRI2965 | AWRI2969 | P value | F-value |
|---|---|---|---|---|---|---|---|---|---|
| Acetic acid (mg L$^{-1}$) | AceAc | 226.8 ± 20.7$^{ab}$ | 191.5 ± 22.4$^{b}$ | 225.5 ± 3.6$^{ab}$ | 237.6 ± 24.5$^{ab}$ | 254.6 ± 20.8$^{a}$ | 276.3 ± 25.4$^{a}$ | 0.006 | 5.7 |
| Ethyl acetate (mg L$^{-1}$) | EthAt | 23.2 ± 0.26$^{b}$ | 25.0 ± 0.44$^{b}$ | 27.3 ± 0.78$^{a}$ | 27.6 ± 1.5$^{a}$ | 25.3 ± 1.7$^{b}$ | 24.2 ± 1.1$^{b}$ | 0.004 | 6.4 |
| 2-methylpropyl acetate | 2MeProAt | 70 ± 18$^{b}$ | 72 ± 24$^{b}$ | 128 ± 18$^{a}$ | 120 ± 15$^{a}$ | 58 ± 3.4$^{b}$ | 72 ± 6.7$^{b}$ | 0.001 | 10.2 |
| 2-methylbutyl acetate | 2MeButAt | 108 ± 5.2$^{d}$ | 160 ± 3.7$^{c}$ | 209 ± 10.2$^{a}$ | 195 ± 5.1$^{a}$ | 147 ± 3.6$^{c}$ | 178 ± 4.3$^{b}$ | <0.001 | 118 |
| 3-methylbutyl acetate | 3MeButAt | 1907 ± 96$^{c}$ | 1918 ± 47$^{c}$ | 2720 ± 52$^{a}$ | 2415 ± 59$^{b}$ | 1940 ± 26$^{c}$ | 2341 ± 14$^{b}$ | <0.001 | 111 |
| 2-PEA | 2PheEthAt | 228 ± 20$^{f}$ | 1096 ± 26$^{e}$ | 7283 ± 250$^{a}$ | 3047 ± 25$^{c}$ | 2703 ± 17$^{d}$ | 3709 ± 37$^{b}$ | <0.001 | 1655 |
| Ethyl 2-methyl propanoate | Et2MePro | 29.6 ± 0.4 | 29.1 ± 1.0 | 31.9 ± 0.8 | 28.9 ± 1.0 | 31.4 ± 2.3 | 31.2 ± 0.8 | 0.030 | 3.7 |
| Ethyl propanoate | EtPro | 25.1 ± 0.4$^{ab}$ | 26.7 ± 0.2$^{a}$ | 25.7 ± 0.2$^{ab}$ | 24.5 ±0.7$^{b}$ | 25.8 ± 0.8$^{ab}$ | 25.2 ± 1.5$^{ab}$ | 0.063 | 2.9 |
| Ethyl 2-methylbutanoate | Et2MeBut | 10.8 ± 0.6$^{b}$ | 11.3 ± 0.1$^{ab}$ | 11.3 ± 0.1$^{ab}$ | 11.0 ± 0.6$^{b}$ | 11.4 ± 0.1$^{ab}$ | 12.2 ± 0.2$^{a}$ | 0.010 | 5.1 |
| Ethyl 3-methylbutanoate | Et3MeBut | 7.2 ± 0.4$^{ab}$ | 7.2 ± 0.2$^{ab}$ | 7.2 ± 0.1$^{ab}$ | 7.0 ± 0.3$^{b}$ | 7.1 ± 0.7$^{ab}$ | 8.1 ± 0.5$^{a}$ | 0.045 | 3.2 |
| Ethyl butanoate | EtBut | 206 ± 7$^{bc}$ | 202 ± 16$^{c}$ | 244 ± 3$^{a}$ | 214 ± 3$^{bc}$ | 208 ± 10$^{bc}$ | 228 ± 6$^{ab}$ | 0.001 | 9.8 |
| Ethyl hexanoate | EtHex | 315 ± 14$^{d}$ | 361 ± 3.4$^{a}$ | 360 ± 9.9$^{a}$ | 327 ± 3.7$^{cd}$ | 337 ± 3.2$^{bc}$ | 356 ± 3.9$^{ab}$ | <0.001 | 19.8 |
| Ethyl octanoate | EtOct | 528 ± 9.8$^{c}$ | 573 ± 6.5$^{b}$ | 602 ± 4.2$^{a}$ | 546 ± 6.9$^{c}$ | 541 ± 8.5$^{c}$ | 585 ± 9.6$^{ab}$ | <0.001 | 40.5 |
| Ethyl decanoate | EtDec | 172 ± 13 | 175 ± 15 | 163 ± 11 | 167 ± 14 | 153 ± 16 | 154 ± 8 | 0.277 | 1.5 |
| Hexyl acetate | HexAt | 215 ± 11$^{d}$ | 218 ± 6.7$^{cd}$ | 269 ± 8.6$^{a}$ | 246 ± 4.9$^{b}$ | 237 ± 2.3$^{bc}$ | 249 ± 4.5$^{b}$ | <0.001 | 25.9 |
| butanol | BtOH | 446 ± 87$^{b}$ | 644 ± 60$^{ab}$ | 671 ± 49$^{a}$ | 556 ± 85$^{ab}$ | 610 ± 20$^{ab}$ | 616 ± 108$^{ab}$ | 0.034 | 3.5 |
| 2-methylpropanol (mg L$^{-1}$) | 2MePrOH | 21.1 ± 2.7$^{c}$ | 25.8 ± 0.61$^{bc}$ | 32.1 ± 0.67$^{a}$ | 27.1 ± 3.6$^{ab}$ | 21.3 ± 2.4$^{bc}$ | 24.1 ± 0.61$^{bc}$ | <0.001 | 11.1 |
| 2-methylbutanol (mg L$^{-1}$) | 2MeBtOH | 29.4 ± 0.74$^{ab}$ | 30.5 ± 0.65$^{ab}$ | 32.9 ± 3.5$^{a}$ | 35.2 ± 3.3$^{a}$ | 26.5 ± 0.75$^{b}$ | 30.1 ± 1.7$^{ab}$ | 0.006 | 5.9 |
| 3-methylbutanol (mg L$^{-1}$) | 3MeBtOH | 103.2 ± 3.6$^{b}$ | 103.3 ± 3.2$^{b}$ | 114.4 ± 6.0$^{a}$ | 114.6 ± 2.4$^{a}$ | 91.3 ± 0.61$^{c}$ | 104.5 ± 2.9$^{b}$ | <0.001 | 18.4 |
| hexanol | HxOH | 1207 ± 46$^{a}$ | 1105 ± 41$^{ab}$ | 1029 ± 40$^{b}$ | 1057 ± 40$^{b}$ | 1121 ± 31$^{ab}$ | 1098 ± 26$^{b}$ | 0.002 | 7.9 |
| 2-PE (mg L$^{-1}$) | 2PhenEtOH | 18.3 ± 2.4$^{e}$ | 47.7 ± 1.7$^{d}$ | 263.8 ± 7.4$^{a}$ | 121.4 ± 2.9$^{c}$ | 138.6 ± 1.9$^{b}$ | 149.0 ± 5.1$^{b}$ | <0.001 | 1320 |
| TyrOH (mg L$^{-1}$) | Tsol | 14.1 ± 1.2$^{b}$ | 11.0 ± 0.4$^{bc}$ | 6.6 ± 0.1$^{c}$ | 8.7 ± 0.1$^{c}$ | 122 ± 4.2$^{a}$ | 8.9 ± 1.4$^{c}$ | <0.001 | 1797 |
| TOL (mg L$^{-1}$) | Tphol | 0.08 ± 0.1$^{b}$ | 0.28 ± 0.1$^{b}$ | 39.2 ± 1.3$^{a}$ | 1.01 ± 0.2 $^{b}$ | 0.98 ± 0.2$^{b}$ | 1.02 ± 0.3$^{b}$ | <0.001 | 2252 |
| TOL-$SO_3$H (mg L$^{-1}$) | Tsol_SO3 | 1.25 ± 0.1$^{e}$ | 3.6 ± 0.1$^{d}$ | 19.9 ± 1.1$^{a}$ | 7.6 ± 0.5$^{c}$ | 9.6 ± 0.2$^{b}$ | 7.2 ± 0.5 $^{c}$ | <0.001 | 298 |

| | | | | | | | | | |
|---|---|---|---|---|---|---|---|---|---|
| Propanoic acid | ProAc | 1077 ± 249 | 1748 ± 529 | 1729 ± 343 | 1349 ± 478 | 1768 ± 229 | 1782 ± 603 | 0.288 | 1.4 |
| Butanoic acid | ButAc | 1211 ± 82 | 1363 ± 110 | 1433 ± 131 | 1216 ± 78 | 1323 ± 85 | 1428 ± 166 | 0.114 | 2.3 |
| 3-methyl butanoic acid | 3MeButAc | 822 ± 34 | 834 ± 6.5 | 861 ± 60 | 859 ± 92 | 807 ± 14 | 846 ± 52 | 0.773 | 0.5 |
| 2-methyl butanoic acid | 2MeButAc | 402 ± 21 | 388 ± 4.9 | 397 ± 23 | 382 ± 6.6 | 394 ± 23 | 397 ± 20 | 0.785 | 0.5 |
| Hexanoic acid | HexAc | 5659 ± 283$^{c}$ | 6661 ± 191$^{ab}$ | 7026 ± 209$^{a}$ | 6071 ± 185$^{bc}$ | 6247 ± 21$^{bc}$ | 6743 ± 493$^{ab}$ | 0.001 | 10.3 |
| Octanoic acid | OctAc | 8024 ± 526$^{b}$ | 8692 ± 259$^{ab}$ | 8957 ± 365$^{ab}$ | 8106 ± 165$^{ab}$ | 8316 ± 381$^{ab}$ | 9000 ± 270$^{a}$ | 0.015 | 4.5 |
| Decanoic acid | DecAc | 1792 ± 92$^{a}$ | 1665 ± 123$^{ab}$ | 1601 ± 36$^{ab}$ | 1659 ± 112$^{ab}$ | 1460 ± 209$^{b}$ | 1.603 ± 27$^{ab}$ | 0.083 | 2.6 |
| Free $SO_2$ (mg $L^{-1}$) | $FSO_2$ | 23 ± 4.4 | 23 ± 3.6 | 23 ± 3.1 | 20 ± 1.0 | 21 ± 4.7 | 18 ± 1.0 | 0.355 | 1.2 |
| Total $SO_2$ (mg $L^{-1}$) | $TSO_2$ | 80 ± 7.6 | 86 ± 4.7 | 85 ± 6.1 | 83 ± 2.9 | 85 ± 8.3 | 76 ± 1.0 | 0.293 | 1.4 |
| $H_2S$ | H2S | 0.4 ± 0.10 | 0.4 ± 0.04 | 0.4 ± 0.05 | 0.4 ± 0.01 | 0.4 ± 0.0 | 0.4 ± 0.02 | 0.362 | 1.2 |
| MeSH | MeSH | 1.54 ± 0.19$^{a}$ | 1.31 ± 0.16$^{ab}$ | 1.19 ± 0.08$^{b}$ | 1.30 ± 0.05$^{ab}$ | 1.28 ± 0.11$^{ab}$ | 1.30 ± 0.12$^{ab}$ | 0.090 | 2.5 |
| DMS | DMS | 3.0 ± 0.1$^{b}$ | 1.8 ± 0.1$^{c}$ | 2.3 ± 0.3$^{c}$ | 2.0 ± 0.2$^{c}$ | 3.5 ± 0.2$^{ab}$ | 3.5 ± 0.1$^{a}$ | <0.001 | 44.3 |
| MeSAc | MeSAc | 5.5 ± 0.4$^{a}$ | 4.2 ± 0.2$^{bc}$ | 4.8 ± 0.1$^{ab}$ | 4.0 ± 0.1$^{cd}$ | 3.5 ± 0.2$^{d}$ | 4.6 ± 0.5$^{bc}$ | <0.001 | 19.1 |
| Methionol | Metol | 542 ± 75$^{d}$ | 671 ± 5$^{cd}$ | 2280 ± 198$^{a}$ | 1164 ± 39$^{b}$ | 537 ± 25$^{d}$ | 794 ± 49$^{c}$ | 0.000 | 163 |
| Methional | Metal | 1.71 ± 0.4$^{a}$ | 1.91 ± 0.2$^{a}$ | 0.84 ± 0.3$^{b}$ | 1.37 ± 0.4$^{ab}$ | 0.84 ± 0.2$^{b}$ | 1.25 ± 0.1$^{ab}$ | 0.001 | 8.5 |
| 2-methylpropanal | 2MeProAl | 5.4 ± 1.6$^{ab}$ | 6.2 ± 0.7$^{ab}$ | 7.3 ± 2.2$^{a}$ | 5.1 ± 1.3$^{ab}$ | 2.9 ± 0.3$^{b}$ | 5.5 ± 1.2$^{ab}$ | 0.042 | 3.3 |
| 3-methylbutanal | 2MeButAl | 4.4 ± 0.9$^{ab}$ | 5.8 ± 0.6$^{a}$ | 4.9 ± 1.3$^{ab}$ | 4.9 ± 0.5$^{ab}$ | 3.0 ± 0.1$^{b}$ | 4.7 ± 1.5$^{ab}$ | 0.066 | 2.8 |
| furfural | Furfal | 12.5 ± 3.6$^{b}$ | 19.6 ± 0.4$^{ab}$ | 38.5 ± 16$^{a}$ | 29.4 ± 7.1$^{ab}$ | 21.8 ± 0.7$^{ab}$ | 24.4 ± 5.4$^{ab}$ | 0.037 | 3.6 |
| 5-methylfurfural | MeFFAL | 1.1 ± 0.4 | 1.5 ± 0.7 | 1.2 ± 0.6 | 1.1 ± 0.4 | 1.4 ± 0.6 | 1.2 ± 0.5 | 0.860 | 0.4 |
| benzaldehyde | Benzal | 61 ± 16 | 65 ± 4.0 | 57 ± 1.5 | 65 ± 8.1 | 55 ± 7.5 | 54 ± 6.4 | 0.448 | 1.0 |
| 2-phenylacetaldehyde | PAceAld | 5.1 ± 1.0$^{c}$ | 6.7 ± 1.3$^{bc}$ | 10.9 ± 0.5$^{a}$ | 9.2 ± 1.2$^{ab}$ | 9.6 ± 1.0$^{ab}$ | 9.1 ± 0.8$^{ab}$ | <0.001 | 11.5 |
| linalool | linool | 2.7 ± 0.4 | 3.7 ± 0.8 | 2.8 ± 0.1 | 3.9 ± 0.6 | 3.5 ± 0.1 | 3.1 ± 0.4 | 0.031 | 3.6 |
| nerol | nerol | 1.9 ± 0.1 | 1.8 ± 0.1 | 1.8 ± 0.2 | 1.8 ± 0.1 | 1.8 ± 0.1 | 1.8 ± 0.3 | 0.894 | 0.3 |
| geraniol | gerol | 3.4 ± 0.1$^{a}$ | 3.3 ± 0.1$^{a}$ | 2.1 ± 0.2$^{b}$ | 1.9 ± 0.1$^{bc}$ | 1.6 ± 0.3$^{c}$ | 2.0 ± 0.1$^{bc}$ | <0.001 | 62 |
| *cis*-rose oxide | RosOx | 1.7 ± 0.1$^{a}$ | 1.6 ± 0.1$^{ab}$ | 1.5 ± 0.1$^{ab}$ | 1.5 ± 0.1$^{ab}$ | 1.4 ± 0.1$^{b}$ | 1.6 ± 0.1$^{a}$ | 0.006 | 5.7 |
| α-terpineol | αTrpol | 2.4 ± 0.1 | 2.4 ± 0.3 | 2.4 ± 0.2 | 2.5 ± 0.1 | 2.4 ± 0.1 | 2.4 ± 0.2 | 0.924 | 0.3 |
| β-damascenone | BDM | 2.6 ± 0.4 | 2.8 ± 0.3 | 2.8 ± 0.2 | 3.3 ± 0.2 | 2.7 ± 0.5 | 3.1 ± 0.1 | 0.132 | 2.1 |

Results are the means ± SD of three independent fermentation replicates. Means with the same superscript letter are not significantly different from each other (Tukey's test, P = 0.05).

**Table S11.** Composition of the wines after 15 months in bottle. Wines were made using AWRI796 (parent strain) and five variants carrying mutations in Aro4p (AWRI2965) or Tyr1p (AWRI2936, 2940, 4124, and 2969). Concentrations of the different metabolites are shown in μg L$^{-1}$, unless otherwise indicated.

| Metabolite) | Label | AWRI796 | AWRI2936 | AWRI2940 | AWRI4124 | AWRI2965 | AWRI2969 | P value | F-value |
|---|---|---|---|---|---|---|---|---|---|
| Acetic acid (mg L$^{-1}$) | AceAc | 434.3 ± 36.8$^{c}$ | 371.8 ± 7.7$^{d}$ | 408.1 ± 28.5$^{cd}$ | 441.9 ± 14.4$^{bc}$ | 505.7 ± 13.6$^{a}$ | 500.4 ± 17.3$^{ab}$ | 0.005 | 16.7 |
| Ethyl acetate (mg L$^{-1}$) | EthAt | 38.1 ± 3.5$^{a}$ | 37.0 ± 0.79$^{ab}$ | 35.1 ± 2.8$^{ab}$ | 36.8 ± 0.80$^{ab}$ | 31.9 ± 1.2$^{b}$ | 30.7 ± 6.5$^{b}$ | 0.010 | 5.0 |
| 2-methylpropyl acetate | 2MeProAt | 28.9 ± 2.6$^{b}$ | 25.7 ± 6.7$^{b}$ | 52.4 ± 10.6$^{a}$ | 48.1 ± 6.4$^{a}$ | 28.6 ± 7.9$^{b}$ | 24.8 ± 2.5$^{b}$ | 0.001 | 9.8 |
| 2-methylbutyl acetate | 2MeButAt | 59 ± 0.1 | 52 ± 13 | 73 ± 15 | 77 ± 14 | 59 ± 12 | 58 ± 4.9 | 0.137 | 2.1 |
| 3-methylbutyl acetate | 3MeButAt | 890 ± 54 | 631 ± 196 | 1,040 ± 288 | 996 ± 276 | 811 ± 231 | 725 ± 75 | 0.203 | 1.7 |
| 2-PEA | 2PheEthAt | 135 ± 12$^{d}$ | 497 ± 129$^{cd}$ | 4270 ± 794$^{a}$ | 1761 ± 394$^{b}$ | 1571 ± 381$^{bc}$ | 1836 ± 21$^{b}$ | <0.001 | 40.1 |
| Ethyl 2-methyl propanoate | Et2MePro | 178 ± 7.8 | 209 ± 24 | 217 ± 26 | 192 ± 27 | 204 ± 29 | 192 ± 26 | 0.469 | 1.0 |
| Ethyl propanoate | EtPro | 89 ± 3.7 | 94 ± 3.3 | 93 ± 5.5 | 92 ± 5.0 | 89 ± 4.3 | 83 ± 9.7 | 0.249 | 1.5 |
| Ethyl 2-methylbutanoate | Et2MeBut | 14.4 ± 0.7 | 19.1 ± 3.4 | 16.5 ± 3.5 | 16.5 ± 4.0 | 16.5 ± 3.8 | 17.8 ± 3.0 | 0.626 | 0.7 |
| Ethyl 3-methylbutanoate | Et3MeBut | 27.3 ± 0.9 | 36.4 ± 7.9 | 33.8 ± 5.9 | 32.1 ± 6.4 | 30.1 ± 4.7 | 37.2 ± 4.3 | 0.283 | 1.4 |
| Ethyl butanoate | EtBut | 329 ± 16$^{b}$ | 331 ± 16$^{b}$ | 389 ± 3.1$^{a}$ | 328 ± 11$^{b}$ | 346 ± 10$^{ab}$ | 333 ± 38$^{b}$ | 0.014 | 4.6 |
| Ethyl hexanoate | EtHex | 646 ± 13$^{c}$ | 744 ± 12$^{ab}$ | 771 ± 10$^{a}$ | 682 ± 2.1$^{c}$ | 699 ± 10$^{bc}$ | 692 ± 50$^{bc}$ | <0.001 | 12.1 |
| Ethyl octanoate | EtOct | 954 ± 20$^{b}$ | 1001 ± 40$^{ab}$ | 1030 ± 21$^{a}$ | 960 ± 18$^{ab}$ | 951 ± 22$^{b}$ | 982 ± 31$^{ab}$ | 0.020 | 4.2 |
| Ethyl decanoate | EtDec | 319 ± 17$^{ab}$ | 297 ± 8.2$^{ab}$ | 293 ± 11$^{ab}$ | 299 ± 24$^{ab}$ | 284 ± 37$^{b}$ | 351 ± 19$^{a}$ | 0.028 | 3.8 |
| Hexyl acetate | HexAt | 97 ± 7.0 | 66 ± 20 | 98 ± 23 | 96 ± 27 | 90 ± 26 | 76 ± 7.2 | 0.338 | 1.2 |
| butanol | BtOH | 733 ± 60$^{b}$ | 1036 ± 103$^{a}$ | 959 ± 111$^{a}$ | 865 ± 42$^{ab}$ | 949 ± 45$^{a}$ | 939 ± 6.0$^{a}$ | 0.004 | 6.4 |
| 2-methylpropanol (mg L$^{-1}$) | 2MePrOH | 27.4 ± 0.56$^{d}$ | 30.4 ± 1.1$^{c}$ | 39.1 ± 0.84$^{a}$ | 35.6 ± 0.21$^{b}$ | 28.2 ± 0.60$^{d}$ | 29.1 ± 0.58$^{cd}$ | <0.001 | 136 |
| 2-methylbutanol (mg L$^{-1}$) | 2MeBtOH | 29.6 ± 1.8$^{b}$ | 35.3 ± 2.0$^{a}$ | 35.1 ± 1.4$^{a}$ | 37.0 ± 2.1$^{a}$ | 29.8 ± 0.91$^{b}$ | 33.4 ± 1.6$^{ab}$ | 0.001 | 9.9 |
| 3-methylbutanol (mg L$^{-1}$) | 3MeBtOH | 144.0 ± 8.4$^{ab}$ | 144.2 ± 8.6$^{ab}$ | 157.4 ± 6.7$^{a}$ | 160.3 ± 8.0$^{a}$ | 131.1 ± 4.3$^{b}$ | 150.1 ± 4.3$^{ab}$ | 0.003 | 6.9 |
| hexanol | HxOH | 1982 ± 27$^{a}$ | 1773 ± 27$^{c}$ | 1753 ± 16$^{c}$ | 1800 ± 21$^{c}$ | 1902 ± 10$^{b}$ | 1802 ± 41$^{c}$ | <0.001 | 35.0 |
| 2-PE (mg L$^{-1}$) | 2PhenEtOH | 26.2 ± 3.2$^{f}$ | 66.6 ± 2.1$^{e}$ | 398.1 ± 7.5$^{a}$ | 177.7 ± 4.3$^{d}$ | 192.9 ± 4.6$^{c}$ | 211.6 ± 2.2$^{b}$ | <0.001 | 2676 |
| TyrOH (mg L$^{-1}$) | Tsol | 15.6 ± 0.7$^{b}$ | 12.0 ± 0.5$^{bc}$ | 6.8 ± 0.3$^{c}$ | 9.8 ± 0.5$^{bc}$ | 124 ± 5.1$^{a}$ | 9.5 ± 1.6$^{bc}$ | <0.001 | 1312 |
| TOL (mg L$^{-1}$) | Tphol | 0.05 ± 0.1$^{c}$ | 0.32 ± 0.1$^{c}$ | 39.7 ± 1.0$^{a}$ | 1.01 ± 0.3$^{bc}$ | 1.63 ± 0.3$^{b}$ | 0.58 ± 0.1$^{bc}$ | <0.001 | 3814 |
| TOL-SO$_3$H (mg L$^{-1}$) | Tsol_SO3 | 1.29 ± 0.1$^{e}$ | 3.68 ± 0.1$^{d}$ | 22.6 ± 2.2$^{a}$ | 8.2 ± 0.7$^{c}$ | 9.5 ± 0.2$^{b}$ | 7.3 ± 0.3$^{c}$ | <0.001 | 79 |

| | | | | | | | | | |
|---|---|---|---|---|---|---|---|---|---|
| Propanoic acid | ProAc | 1140 ± 102 | 1019 ± 252 | 1006 ± 249 | 925 ± 92 | 1066 ± 43 | 1080 ± 124 | 0.696 | 0.6 |
| Butanoic acid | ButAc | 846 ± 70$^{c}$ | 1100 ± 26$^{a}$ | 1085 ± 80$^{ab}$ | 901 ± 88$^{bc}$ | 1002 ± 75$^{ab}$ | 1135 ± 71$^{a}$ | 0.002 | 8.0 |
| 3-methyl butanoic acid | 3MeButAc | 645 ± 7$^{ab}$ | 638 ± 7$^{b}$ | 654 ± 13$^{ab}$ | 576 ± 9$^{c}$ | 637 ± 15$^{b}$ | 683 ± 30$^{a}$ | <0.001 | 15.2 |
| 2-methyl butanoic acid | 2MeButAc | 371 ± 8 | 356 ± 30 | 352 ± 25 | 342 ± 15 | 378 ± 24 | 378 ± 35 | 0.397 | 1.1 |
| Hexanoic acid | HexAc | 7048 ± 397$^{c}$ | 8205 ± 316$^{abc}$ | 8636 ± 490$^{a}$ | 7408 ± 535$^{abc}$ | 7271 ± 354$^{bc}$ | 8421 ± 626$^{ab}$ | 0.005 | 6.2 |
| Octanoic acid | OctAc | 10423 ± 135$^{bc}$ | 11110 ± 302$^{a}$ | 11366 ± 229$^{a}$ | 10405 ± 44$^{bc}$ | 10261 ± 174$^{c}$ | 10942 ± 208$^{ab}$ | <0.001 | 15.3 |
| Decanoic acid | DecAc | 2221 ± 80$^{a}$ | 1942 ± 134$^{ab}$ | 1934 ± 124$^{ab}$ | 1965 ± 134$^{ab}$ | 1858 ± 161$^{b}$ | 1857 ± 70$^{b}$ | 0.030 | 3.7 |
| Free $SO_2$ (mg $L^{-1}$) | $FSO_2$ | 15 ± 2.0 | 15 ± 4.9 | 17 ± 3.1 | 10 ± 0.6 | 14 ± 3.5 | 11 ± 0.6 | 0.128 | 2.2 |
| Total $SO_2$ (mg $L^{-1}$) | $TSO_2$ | 72 ± 4.9 | 80 ± 8.3 | 80 ± 7.4 | 69 ± 1.0 | 77 ± 6.7 | 68 ± 1.5 | 0.099 | 2.4 |
| $H_2S$ | H2S | 3.5 ± 0.9 | 3.6 ± 1.0 | 3.0 ± 1.1 | 4.0 ± 1.2 | 2.3 ± 0.9 | 1.8 ± 0.3 | 0.101 | 2.4 |
| MeSH | MeSH | 8.7 ± 0.8$^{a}$ | 7.6 ± 0.5$^{ab}$ | 7.4 ± 0.3$^{ab}$ | 7.8 ± 0.2$^{a}$ | 4.6 ± 0.5$^{c}$ | 6.3 ± 0.6$^{b}$ | <0.001 | 22.1 |
| DMS | DMS | 15.9 ± 0.8$^{b}$ | 16.4 ± 1.9$^{b}$ | 16.5 ± 0.9$^{b}$ | 14.5 ± 0.5$^{b}$ | 26.9 ± 0.5$^{a}$ | 26.9 ± 0.4$^{a}$ | <0.001 | 106 |
| MeSAc | MeSAc | nd | nd | nd | nd | nd | nd | | |
| Methionol | Metol | 567 ± 57$^{c}$ | 679 ± 20$^{c}$ | 2102 ± 205$^{a}$ | 1104 ± 34$^{b}$ | 571 ± 12$^{c}$ | 807 ± 17$^{c}$ | <0.001 | 133 |
| Methional | Metal | 26.1 ± 2.9$^{ab}$ | 28.0 ± 6.8$^{a}$ | 12.9 ± 3.1$^{c}$ | 29.4 ± 1.9$^{a}$ | 15.9 ± 3.7$^{c}$ | 19.9 ± 2.2$^{bc}$ | 0.001 | 9.8 |
| 2-methylpropanal | 2MeProAl | 128 ± 12 | 151 ± 31 | 153 ± 38 | 127 ± 2.7 | 128 ± 8.3 | 136 ± 7.1 | 0.477 | 1.0 |
| 3-methylbutanal | 2MeButAl | 26.3 ± 0.4$^{c}$ | 35.9 ± 3.3$^{b}$ | 43.4 ± 4.1$^{a}$ | 40.4 ± 1.3$^{ab}$ | 43.7 ± 1.6$^{a}$ | 40.5 ± 1.4$^{ab}$ | <0.001 | 22.7 |
| furfural | Furfal | 257 ± 31 | 226 ± 14 | 222 ± 26 | 259 ± 4.5 | 236 ± 35 | 248 ± 24 | 0.361 | 1.2 |
| 5-methylfurfural | MeFFAL | 7.7 ± 0.1$^{a}$ | 6.7 ± 0.7$^{ab}$ | 5.5 ± 0.4$^{b}$ | 6.7 ± 0.4$^{ab}$ | 6.7 ± 0.5$^{ab}$ | 6.0 ± 0.9$^{ab}$ | 0.021 | 4.3 |
| benzaldehyde | Benzal | 130 ± 18 | 152 ± 17 | 176 ± 39 | 142 ± 7.7 | 158 ± 16 | 158 ± 26 | 0.283 | 1.4 |
| 2-phenylacetaldehyde | PAceAld | 25.8 ± 1.0$^{e}$ | 35.3 ± 1.6$^{d}$ | 107 ± 4.2$^{a}$ | 60.7 ± 1.7$^{c}$ | 83.9 ± 3.1$^{b}$ | 63.4 ± 3.2$^{c}$ | <0.001 | 373 |

Results are the mean ± SD of three independent fermentation replicates (μg $L^{-1}$). Means with the same superscript letter are not significantly different from each other (Tukey's test, P = 0.05). n.d.: not detected

**Figure S1**

Relationship between 2-PE and 2-PEA production at the end of alcoholic fermentation in the Chardonnay wines. The wines were fermented by the parent AWRI796 (●) strain, and five variants carrying mutations in Aro4p (AWRI2965 (●) or Tyr1p (AWRI2936 (●), AWRI2940 (●), AWRI4124 (●) and AWRI2969 (●)). Results are the mean (in mg $L^{-1}$) of three independent fermentation replicates.

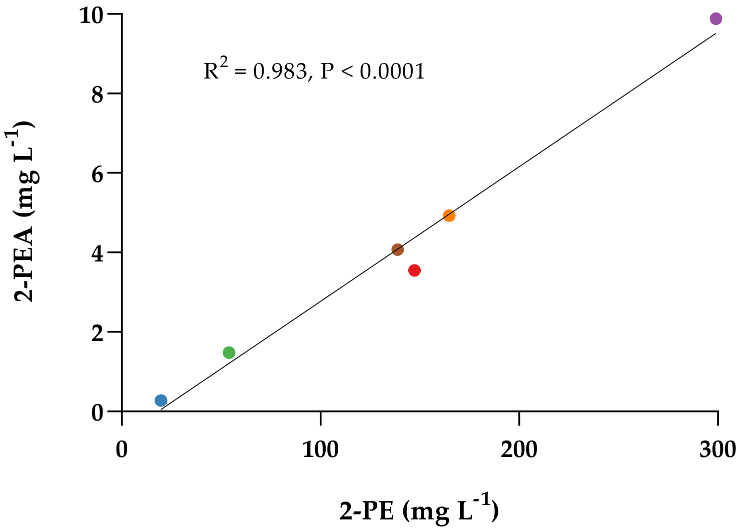

**Figure S2**

Relationship between concentrations of TOL (in mg $L^{-1}$) at the end of alcoholic fermentation and its maximum percentage of conversion into TOL-$SO_3H$ in the Chardonnay wines during ageing. Wines were fermented by the parent AWRI796 (●) strain, or five variants carrying mutations in Aro4p (AWRI2965 (●) or Tyr1p (AWRI2936 (●), AWRI2940 (●), AWRI4124 (●) and AWRI2969 (●)). The graph is zoomed-in to highlight the relationship between these two variables at low to moderate concentrations of TOL (< 10 mg $L^{-1}$). Results are the mean of three independent fermentation replicates.

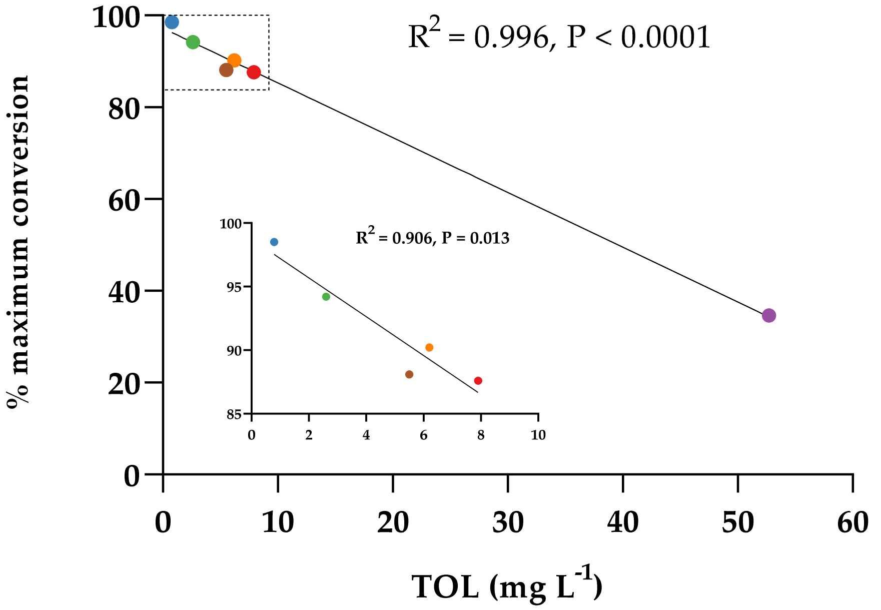

## Figure S3

Relationship between the decrease in free (filled symbols) and total (empty symbols) $SO_2$ concentrations and TOL-$SO_3H$ formation in the Chardonnay wines at different time points during ageing. Wines were fermented with either the parent AWRI796 (●) strain, and five variants carrying mutations in Aro4p (AWRI2965 (●) or Tyr1p (AWRI2936 (●), AWRI2940 (●), AWRI4124 (●) and AWRI2969 (●)). Results are expressed as the average of three independent triplicates. No correlation was observed between either free or total $SO_2$ and TOL-$SO_3H$ levels in the wines at any given time point ($P < 0.05$).

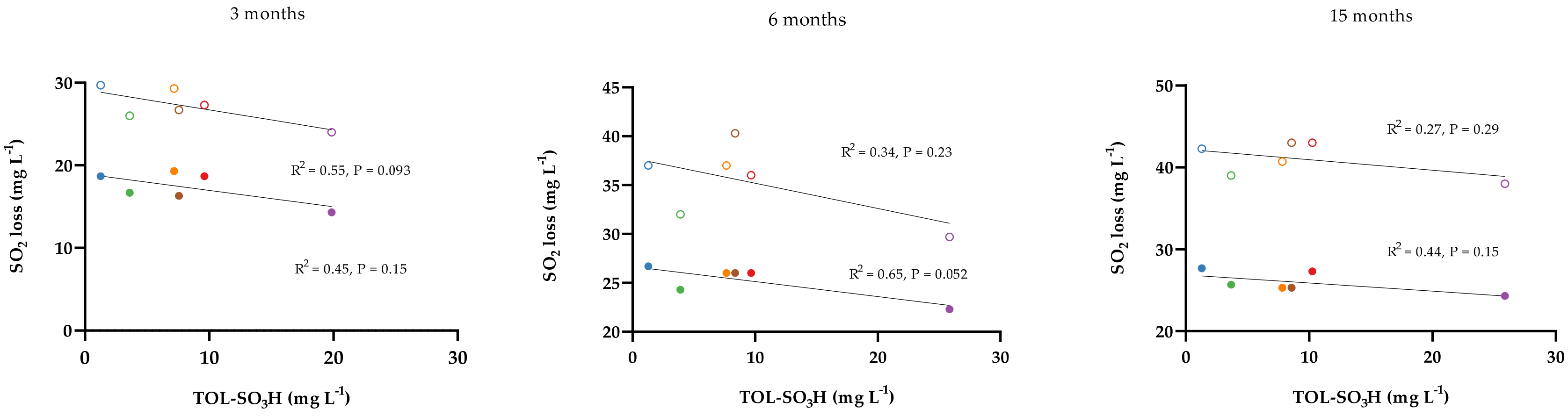

**Figure S4**

Mean blue fluorescence by flow cytometry for AWRI796 and two variants with an altered Ehrlich pathway (AWRI2940 and AWRI2965), with genomic integration of the blue fluorescence protein (BFP) under the control of the *S. cerevisiae ARO9* promoter (Aro9pr). Results are the average of the mean blue fluorescence for three independent replicates after growth for 48 hours in liquid minimal media.

A DNA fragment of 3.4 kb was amplified by PCR from the pCV2-HO-Aro9pr-BFP plasmid, and approximately 200 µg of the purified DNA used to transform each of the different yeast strains. Transformants were selected in YPD-CloNAT plates, and genomic integration in the HO locus was confirmed by PCR. Mean blue fluorescence was acquired after growth in 96-well plates containing 600 µl of Yeast Nitrogen Base medium without amino acids and 2% glucose. Microplates were sealed with Breathe-Easy sealing membranes and incubated at 28°C for 48 hours. Cell cultures were then diluted in PBS buffer to ensure a concentration of about $5x10^5$ cells/ml. Mean blue fluorescence was detected using a violet (405 nm solid state laser) excitation and a 448/50 nm detection filter by flow cytometry and using a Guava easyCyte 12HT instrument. The mean blue fluorescence of the transformed cells was subtracted from that obtained from untransformed cells. For all analysis, a minimum of 5000 events were acquired while cell throughput was kept under 500 cells/µL. Results were analysed with inCyte software version 3.2.

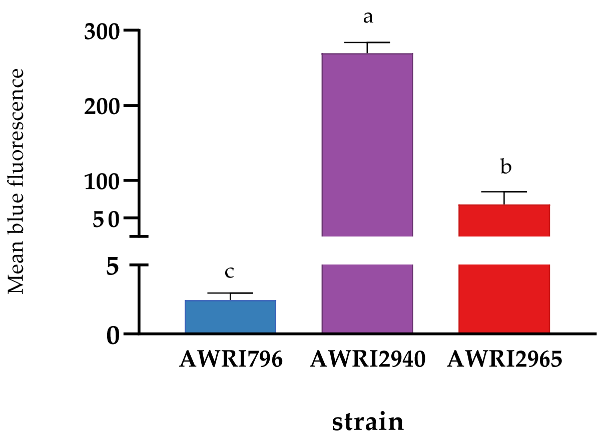

Supplement: Supplementary file 1 [file molecules-26-04979-s001.zip › molecules-1302897-supplementary.pdf]
